# The long-term impact of neonatal hypoxic-ischemic brain injury on neural dynamics in deep-layer retrosplenial cortex and hippocampus in mice

**DOI:** 10.1016/j.isci.2026.115003

**Published:** 2026-03-28

**Authors:** Lida Du, Meng Yang, Hendrik W. Steenland, Zhengwei Luo, Andrea Ovcjak, Ruiyan Hu, Shuzo Sugita, Kaori Takehara-Nishiuchi, Luka Milosevic, Zhong-Ping Feng

**Affiliations:** 1 Department of Physiology, University of Toronto, Toronto, ON, Canada; 2 Neurotek Incorporated, York, ON, Canada; 3 Department of Psychology, University of Toronto, Toronto, ON, Canada; 4 Institute of Biomedical Engineering, University of Toronto, Toronto, ON, Canada

**Keywords:** neuroscience, behavioral neuroscience, sensory neuroscience

## Abstract

Perinatal hypoxic-ischemia (HI) often results in hypoxic-ischemic encephalopathy (HIE), a major cause of neonatal mortality and long-term neurodevelopmental deficits. While motor and cognitive impairments are common, the underlying neuronal mechanisms remain poorly understood. Using a mouse model of neonatal HI, we examined long-term effects on neuronal activity in the retrosplenial cortex (RSC) and hippocampus through simultaneous electrophysiological recordings in freely moving adults. HI mice showed a significant reduction in neurons recorded per tetrode, and remaining pyramidal neurons in the ipsilateral hemisphere exhibited abnormal bursting, synchrony, and pathological spike-field coupling to theta-alpha oscillations. These neural abnormalities coincided with impaired motor learning performance across training sessions. Our findings reveal persistent disruptions in firing patterns and RSC-hippocampus communication, providing insight into long-term motor learning deficits at the single-neuron level, following neonatal HI brain injury.

## Highlights

• Neonatal HI injury causes lasting abnormal firing and synchrony in RSC and hippocampus
• Ipsilateral pyramidal neurons show pathological spike-field coupling to theta-alpha
• Disrupted RSC-hippocampal communication drives long-term motor-learning deficits

## Graphical abstract

**Figure:**
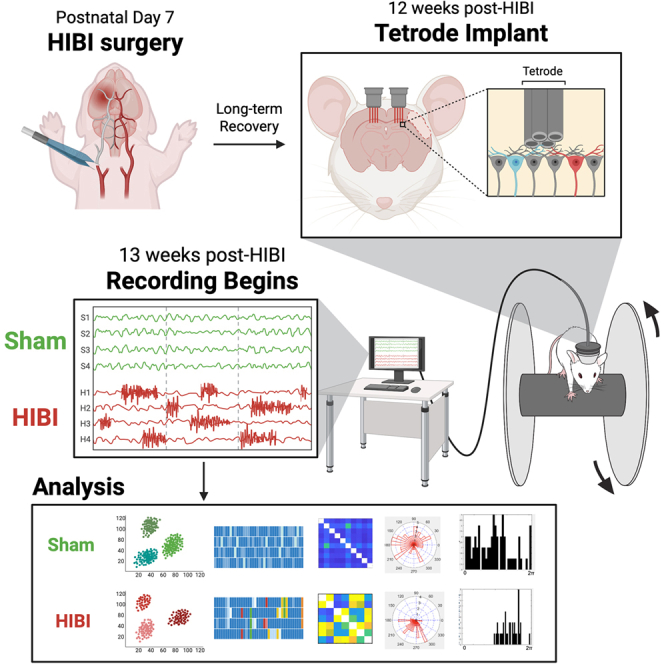

## Introduction

Neonatal hypoxic-ischemic (HI) brain injury results from insufficient oxygen and blood supply to various structures of the neonate’s brain, leading to brain infarction.^1^ This condition often progresses to neonatal hypoxic-ischemic encephalopathy (HIE), a highly prevalent neurological disorder in infants that is associated with long-term neurodevelopmental delay, motor impairment, and learning disabilities.^2^^,^^3^ The incidence of HIE is approximately 0.6% of births in the United States and 2.6% in developing countries.^4^ Notably, around 25% of HIE cases result in infant mortality, while 50%–80% of survivors experience permanent neurological dysfunction.^5^ Clinical studies have shown that infants with mild HIE exhibit lower cognitive composite scores compared to controls, while no significant difference in cognitive scores was observed between untreated children with mild HIE and those with moderate HIE who received therapeutic hypothermia.^6^

Motor impairments after neonatal brain injury are commonly observed and exhibit varied dysfunctions in motor planning, learning, and coordination. Over half of children who have experienced neonatal HIE show motor learning impairments such as difficulties with coordination, motor control, and processing speed.^7^^,^^8^ These additional neuromotor deficits may be underestimated because they often do not become apparent until preschool or school age.^9^ Nevertheless, both basic and clinical evidence show that motor impairments are comparable in patients and mouse models.^10^ However, the long-term impact of HI on brain morphology and function, as well as the mechanisms underlying these changes, is not fully understood. In clinical HIE patients, increasing HIE severity alters both local and large-scale cortical activity, including abnormal burst activity and synchrony.^11^ HIE patients have shown reduced electro-encephalography (EEG) coherence across brain regions, which corresponds with impaired brain function.^12^ Clinical EEG recordings of patients have also shown that theta (4–8 Hz) and alpha (8–12 Hz) oscillations are strongly correlated with navigational and spatial performance, which is associated with retrosplenial cortex (RSC) and hippocampal function.^13^^,^^14^^,^^15^ Understanding HI-affected cortical-hippocampal networks is critical for preventing survivors from suffering permanent neurological dysfunction.

In principle, HI insult directly induces brain infarction in various interconnected cortical and hippocampal regions, thereby affecting their neuronal activities. Early studies on animal models of HI brain injury have reported morphological impairments and the molecular mechanisms responsible for neuronal death in cortical-hippocampal networks.^1^^,^^16^ Recently, HI-induced brain impairments in animal models have been associated with the pathological suppression of oscillatory waves across various frequency bands.^17^^,^^18^ Given their anatomical and functional connectivity, the RSC and hippocampus form a key memory-navigation circuit that interfaces with motor regions via rhythmic oscillations in the theta and alpha bands.^19^^,^^20^ These properties make them well-suited for investigating long-term network dysfunction after neonatal HI.

In electrocorticographic or EEG recordings, neonatal HI brain injury in animal models has been associated with long-term impacts on sleep-wake patterns and low-frequency (0–25 Hz) oscillations, as well as bilateral tonic-clonic activation of the motor cortical and hippocampal circuits and seizure behaviors.^21^^,^^22^ Elucidating HIE-associated neuronal activity changes may aid in stratifying the long-term impacts of HIE.

Studies in rodents have highlighted the interconnected roles of the RSC, hippocampus, and motor cortices in motor skill acquisition.^23^^,^^24^ Specifically, monosynaptic excitatory connections between pyramidal neurons (PNs) in the RSC and the posterior secondary motor cortex across cortical layers contribute to sensorimotor integration and motor control.^23^ As integral components of the motor cortex network, lesions in the RSC or hippocampus significantly impair patterned motor and spatial learning.^25^^,^^26^ In animal studies, HI brain injury directly disrupts the structure and function of developing hippocampal-prefrontal cortex networks^27^ and impairs hippocampal dendritic development.^28^^,^^29^ While both interneurons (INs) and PNs play essential roles in the cortico-hippocampal network, PNs demonstrate higher vulnerability to ischemic and hypoxic conditions *in vitro.*^28^^,^^29^ Although HI brain injury is associated with infarction of the RSC and hippocampus, as well as motor learning deficits, the specific relationship between pathological brain activity in the RSC-hippocampal circuitry and HI-induced behavioral impairments remains unclear.

Also in animal models, HI conditions have shown severe impacts on long-term neurobehavioral outcomes, including motor and cognitive functions, as well as effects on the neural circuitry of the neocortex, striatum, and hippocampus.^30^^,^^31^ It has been suggested that lesions in various subcortical regions, such as the hippocampus and basal ganglia, modulate cerebral functions and, in turn, influence neurobehavioral outcomes.^32^^,^^33^ Beta band oscillations (13–30 Hz) are critical for motor control, and excessive synchronization of beta oscillation is associated with movement disorders.^34^ However, how neonatal HI affects long-term cerebral neuronal activity and oscillations remains unclear.

In this study, we used single-cell recordings in a mouse model of HI to identify specific neuronal types that are critical in the RSC-hippocampal network and to investigate changes in the firing properties and functions of these neurons following HI brain injury. Our study aimed to demonstrate the long-term impacts of neonatal HI brain injury on neuronal firing patterns and oscillatory activity in the RSC and hippocampus, which potentially correlated with motor function impairments, specifically motor learning deficits after neonatal HI brain injury.

## Results

### Neonatal HI insult induces short- and long-term damage in brain morphology

**Figure 1:**
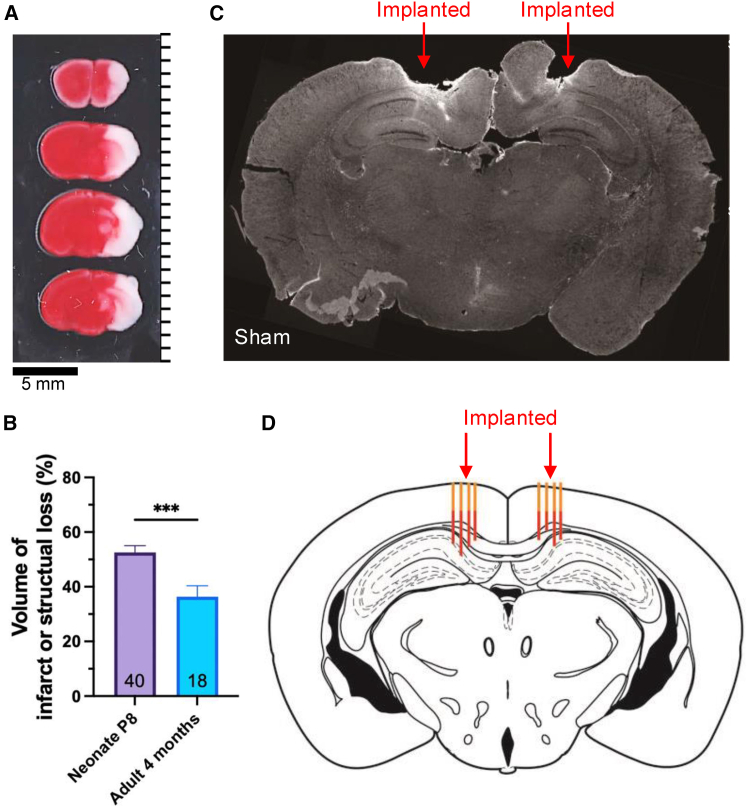
Neonatal HI associated long-term impacts on brain morphology and reconstruction of electrode placement (A) Representative TTC staining showing neonatal HI induced brain infarction of the ipsilateral hemisphere on postnatal day 8, 24 h post- HI (Scale bars, 5 mm). (B) Quantitative graph showing the average percentage of infarction volume/structural loss in neonatal P8 and adult 4-month-old mice. The HI mice at 4-month-old showed recovery with significantly (*p* < 0.01) reduced infarction volume (36.3% ± 4.0%) than that in P8 mice (52.55% ± 2.48%) (Scale bars, 1 mm). (C) Coronal brain sections from 4-month-old sham (top) and HI (bottom) mice, showing the long-term morphological impact of HI on cortices and the hippocampus. Red arrows in the yellow box indicate the location of the implanted tetrodes. (D) Reconstructed location of implanted tetrodes in sham (top) and HI (bottom) mice. The original anterior-posterior (AP) and medial-lateral (ML) locations of each implanted tetrode are indicated with orange lines. During the experiment, the tetrodes were lowered ∼60–120 microns every day along the dorsal-ventral (DV) plane, with the tip of red lines indicating the final position of each tetrode. Recordings were conducted daily for approximately 10–14 days before lowering the tetrodes into the final position. (∗∗, *p* < 0.01; unpaired *t* test) See also Figure S1.

At 24 h following HI brain injury, 2,3,5-triphenyltetrazolium chloride (TTC) staining of coronal brain sections was used to examine the infarcted volume of the brain. Figure 1A shows a representative example of TTC staining, with infarcted brain regions indicated as white areas. The average infarction volume in the HI group was 52.55% ± 2.48% (*n* = 40), with morphological damage observed in both cortical and hippocampal regions of the ipsilateral hemisphere. Recovery at 1, 3, and 7 days after HI (i.e., postnatal days P8, P10, and P14) was assessed using short-term behavioral tests, including the geotaxis reflex, cliff avoidance test, and grip test.^35^^,^^36^^,^^37^ Neurobehavioral performances were compared between sham animals (*n* = 8) and HI animals (*n* = 12). Compared to sham pups, HI-injured pups showed significantly impaired performance across all behavioral tests at each time point (Figure S1). Grip test performance was markedly reduced, reflecting impaired forelimb strength and endurance (*p* < 0.01, two-Way ANOVA with Sidak’s multiple comparison; Figure S1A). Specifically, HI pups demonstrated longer latencies in the geotaxis and cliff avoidance tests (*p* < 0.05, two-Way ANOVA with Sidak’s multiple comparison; Figures S1B and S1C), indicating deficits in vestibular, proprioceptive, and impulse control functions. These behavioral tests confirm that neonatal HI results in significant short-term sensorimotor deficits compared to sham controls.

At long-term observation, 4-month-old HI mice showed an average of 36.3% ± 4.0% (*n* = 18) of brain hemispheric structural loss on coronal sections, indicating the long-term impact of HI-associated brain infarction (Figure 1B). At this time point, a custom hyperdrive was positioned over the cortex to record extracellular unit activity in the RSC and hippocampus using tetrodes.

Extracellular neuronal activities were recorded from the RSC and hippocampal CA1 neurons in two sham and three HI-induced mice via tetrode arrays. Animals remained conscious and were able to move freely in their home cages after tetrode array implantation. Mice were then sacrificed after undergoing the recording procedure, and their brains were cryo-sectioned to verify the recording positions. A representative histological image from the sham group showed intact brain structure and the location of the tetrodes (Figure 1C). Unlike sham brains, adult mice that underwent HI modeling at the neonatal stage showed clearly shrunken or missing brain regions, including the ipsilateral cingulate and lateral cortices and hippocampus. The tetrode locations in Figure 1C corresponded with our surgical records, which traced *in vivo* neuronal and field activities of the RSC and hippocampal CA1 region. Relative to the implantation location, tetrodes were lowered 60–120 microns daily, approximately 30 min before recording sessions, to acquire signals from multiple firing units. The trajectory of each tetrode is illustrated in Figure 1D as red tracks. Multi-channel signals were acquired from eight tetrodes on each recording day.

### Spike sorting and classification identify putative pyramidal neurons and interneurons in the RSC and hippocampus

**Figure 2:**
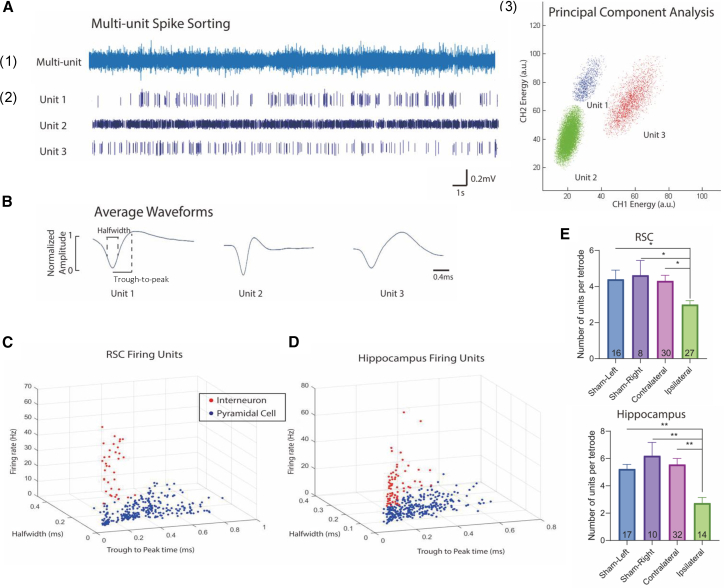
Electrophysiological classification of putative pyramidal neurons and putative interneurons (A) (1) Electrophysiological recording containing unit spiking activities from a single electrode. (2) Principal component analysis (PCA) was applied to identify the multiunit activities into individual unit activities. Spikes from each firing unit were clustered on the energy plot. (3) Spike raster plots for three representative firing units. (B) The three panels correspond to three units sorted by PCA analysis and show the average waveform of the three recorded spiking units. Once sorted, the half-width and the trough-to-peak time values of the waveform were determined. (C and D) Scatterplot of all trough-to-peak times, half-widths, and spiking frequencies for each individual spiking unit (neurons) recorded from the (C) RSC and (D) hippocampus. In the RSC, neurons appeared to form clusters following different patterns; neurons with <0.4 ms trough-to-peak time and >15 Hz frequency were classified as INs.^38^^,^^39^ In the hippocampus, neurons with <0.25 ms trough-to-peak time and >15 Hz frequency were classified as INs.^40^^,^^41^ (E) The number of neurons recorded on the ipsilateral tetrode is significantly (*p* < 0.05 and *p* < 0.01, for the RSC and hippocampus, respectively) reduced that on sham and contralateral hemispheres. (∗, *p* < 0.05, ∗∗, *p* < 0.01; One-way ANOVA with Tukey’s test).

Spike activities from the RSC and hippocampus were recorded by each tetrode as multi-unit signals and sorted into single units based on the electrophysiological characteristics of spike waveform features on each channel of the tetrode. A representative multi-unit signal recorded from three neurons is shown in Figure 2A(1), and its corresponding sorted single-unit spiking activities of each neuron are displayed as raster plots in Figure 2A(2). Spikes that had similar waveforms in the principal-component analysis (PCA) were considered to originate from a single firing unit. Conversely, isolated clusters in the PCA indicated that the spikes were distinct from each other and were classified as different firing units. Representative PCA clusters of three distinct firing units are shown in Figure 2A(3). Distinct firing units with significant action potentials were then identified as putative neurons, which were further classified as narrow- or wide-spiking units, as firing units tended to form clusters above or below a certain trough-to-peak time threshold.^38^ Figure 2B shows three representative firing units with narrow-spiking (spikes with short trough-to-peak time) or wide-spiking (spikes with long trough-to-peak time) features.

In the RSC, two major neuronal firing classes were preliminarily identified based on narrow- or wide-spiking properties and firing rate. We observed that one type of neuron exhibited a relatively long trough-to-peak time (>0.4 ms) with a low spontaneous firing rate (<15 Hz), which was presumed to be PNs. Compared with PNs, presumed INs were characterized by a shorter trough-to-peak time (<0.4 ms) and a faster firing rate (>15 Hz).^38^^,^^39^ Putative PNs and INs were then plotted in Figure 2C based on the trough-to-peak time, firing rate, and half-width features of each individual recorded firing unit. The distinct narrow- or wide-spiking properties and low or high firing rates also allowed us to distinguish between the two classes of neurons in the hippocampal CA1 region. We observed that putative PNs identified in the hippocampus exhibited a relatively long trough-to-peak time (>0.25 ms) with a low spontaneous firing rate (<15 Hz), while presumed INs were characterized by a shorter trough-to-peak time (<0.25 ms) and a faster firing rate (>15 Hz) (Figure 2D).^40^^,^^41^ As mentioned previously, multi-channel signals were acquired from eight tetrodes on each recording day, and tetrodes were lowered 60–120 microns before recording sessions to acquire signals from multiple firing units.

In total, 726 individual PNs and INs were identified in the RSC and hippocampus from two sham (left and right hemispheres) and three HI mice (contralateral and ipsilateral hemispheres) throughout all recording sessions, as summarized in Table 1. Notably, we found that the number of neurons recorded in the RSC and hippocampus of the injured ipsilateral hemisphere in HI mice was significantly reduced compared to controls. Specifically, neuronal counts were reduced by 49.4% and 52.4% compared to the left and right hemispheres of sham mice, respectively, and by 55.9% compared to the contralateral hemisphere of HI mice (*p* < 0.05, One-way ANOVA with Tukey’s test; Figure 2E).

**Table 1:** The number of PNs and INs from bilateral brain hemispheres of sham and HI mice

Treatment Group	Hemisphere	RSC				Hippocampus			
		Pyramidal Cells		Interneurons		Pyramidal Cells		Interneurons	
Sham	left	49	89.1%	6	10.9%	53	82.8%	11	17.2%
	right	57	88.2%	11	11.8%	75	82.4%	16	17.6%
HI	contralateral	120	90.2%	13	9.8%	158	89.8%	18	10.2%
	ipsilateral	89	90.8%	9	9.2%	35	85.4%	6	14.6%

In the sham right and left brain regions, and HI contralateral and ipsilateral brain regions, approximately 90% (*n* = 636) of all recorded neurons (*n* = 726) were PNs. In the ipsilateral hemisphere of HI mice, there was a reduction in the number of PNs and INs in both the RSC and hippocampus in comparison to the contralateral hemisphere.

### Neonatal HI brain injury alters the firing frequency and patterns of cortical and hippocampal pyramidal neurons

**Figure 3:**
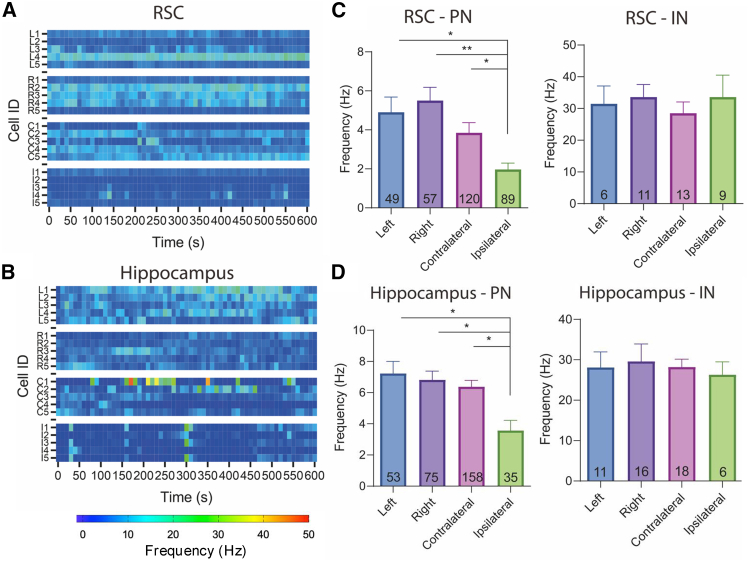
The long-term impact of HI on neuronal firing profiles (A) Firing profiles of typical RSC neurons during freely moving conditions. Neurons in the injured ipsilateral hemisphere (I1-I5) have a reduced firing frequency compared to sham (L1-L5 and R1-R5) and contralateral (C1-C5) neurons. Synchronized firing activities were also observed. (B) Firing profiles of typical hippocampal neurons during freely moving conditions, ipsilateral neurons (I1-I5) have a reduced firing frequency compared to sham (L1-L5 and R1-R5) and contralateral (C1-C5) neurons. Significant synchronized firing activities were observed. (C) Comparison of the average firing frequency of PNs and INs in the RSC. Ipsilateral PNs showed a significantly (*p* < 0.01 for ipsilateral PNs compared with PNs in the right hemisphere in the RSC, *p* < 0.05 for all other comparisons) lower firing frequency compared to contralateral and sham PNs, and no difference in IN firing frequencies was observed. (D) Comparison of the average firing frequency of PNs and INs in the hippocampus. Ipsilateral PNs showed a significantly lower firing frequency compared to contralateral and sham PNs, and there was no difference in IN firing frequencies. (∗, *p* < 0.05, ∗∗, *p* < 0.01; One-way ANOVA with Tukey’s test) see also Figures S2 and S3.

The impact of neonatal HI brain injury on the firing frequency of PNs in the RSC and hippocampus was examined while animals were freely moving in their home cages during recordings. Heatmaps representing the firing frequency of individual representative neurons from the left hemisphere (L1-L5), right hemisphere (R1-R5), contralateral hemisphere (C1-C5), and ipsilateral hemisphere (I1-I5) of the RSC (Figure 3A) and hippocampus (Figure 3B) revealed synchronized neuronal activity in which neurons fired together, an effect not observed in sham mice.

Compared to the left and right RSC of sham mice and the contralateral RSC of HI mice, the average spontaneous firing frequency of PNs in the ipsilateral RSC of HI mice was significantly reduced (*p* < 0.01 for ipsilateral PNs vs. PNs in the right RSC; *p* < 0.05 for all other comparisons, One-way ANOVA with Tukey’s test; Figure 3C). Similarly, the firing frequency of PNs in the ipsilateral hippocampal CA1 region was significantly reduced (*p* < 0.05, One-way ANOVA with Tukey’s test) compared to the left and right hippocampus of sham mice and the contralateral hippocampus of HI mice (Figure 3D).

However, the firing frequency of INs did not show significant changes following neonatal HI brain injury. In addition, synchronized activity in the RSC and hippocampus was consistently observed when individual animals were analyzed separately (Figure S2). Heatmaps from one representative sham and one representative HI mouse revealed that the ipsilateral RSC of the HI mouse did not show an obvious reduction in firing frequency compared to the left and right RSC of the sham mouse and the contralateral RSC of the HI mouse. In contrast, the firing frequency of the ipsilateral hippocampus was reduced compared to the left and right hippocampus of the sham mouse and the contralateral hippocampus of the HI mouse. Interestingly, the contralateral hemisphere in the HI mouse showed complementary activity in the RSC and hippocampus compared with firing frequencies observed in the left and right hemispheres of the sham mouse.

In addition to the long-term impact of HI brain injury on neuronal firing frequency, we found that the firing patterns of PNs were also altered. All identified neurons were classified as “bursting”, “irregularly spiking”, “regularly spiking PN”, or “regularly spiking IN” based on their autocorrelation profiles, as described in a previous study^38^ (Figure S3A). In the ipsilateral RSC, we observed an increased percentage of bursting neurons and a decreased percentage of regularly spiking PNs (Figure S3B). A similar decrease in the percentage of regularly spiking PNs was also observed in the ipsilateral hippocampal CA1 region (Figure S3C). Based on these findings, we focused specifically on the bursting activity of PNs in the RSC and hippocampus across each hemisphere of sham and HI mice, aiming to characterize how HI insult alters PNs firing patterns.

**Figure 4:**
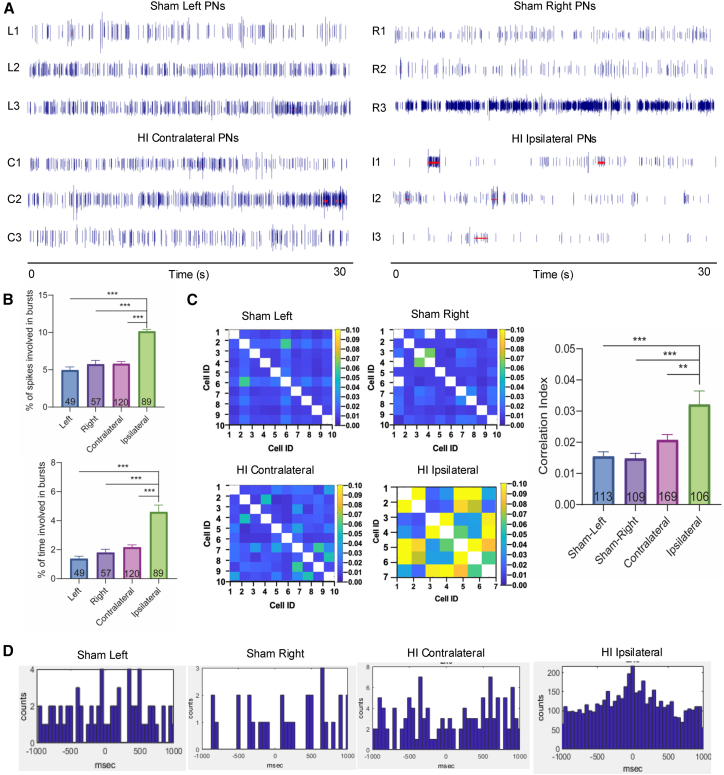
Neonatal HI reduced the firing rate and increased the coherence of cortical neurons (A) Representative example of raster plots showing the neuronal discharge patterns of RSC PNs from the right and left hemispheres of sham mice, and the contralateral and ipsilateral hemispheres of HI mice. Random discharge patterns of RSC PNs were found in sham mice and the HI contralateral hemisphere. In contrast, the firing pattern of the RSC PNs in the HI ipsilateral hemisphere was characterized by increased burst discharges. The bursting activity was defined by the Legendy surprise method^42^: negative logarithm of the firing probability in a random spike with the Poisson surprise threshold at 5. Neuronal bursts are highlighted by red lines. (B) The analyses of the % of total number of spikes in burst discharge (left) and the % of time spent in burst discharge (right) were significantly (*p* < 0.001) increased by HI insult. (C) Ipsilateral PNs in cross-correlation diagrams showed significant (*p* < 0.01 for ipsilateral vs. contralateral; *p* < 0.001 for ipsilateral vs. sham left and right) increases in coherence that suggested synchronized neuronal activities. (D) Cross-correlograms of cell pairs in the RSC suggested that cell pairs from sham and the HI contralateral hemispheres had weakly correlated spike times, leading to dispersive spike time peaks. The HI ipsilateral hemisphere, however, exhibited strong synchronization between neuronal pairs with a sharp peak centered at zero msec. (∗∗, *p* < 0.01, ∗∗∗, *p* < 0.001; One-way ANOVA with Tukey’s test) see also Figures S4–S6.

Neuronal bursting activity was identified by increasing episodes and duration of firing, as defined by the Legendy surprise method.^42^ The spiking patterns of PNs were visualized using raster plots. Representative raster plots of RSC PNs from each experimental group are shown in Figure 4A, with bursting activities marked by horizontal red lines. By analyzing the percentage of total spikes occurring in burst discharges and the percentage of time spent in bursts, we found a significant increase in bursting activity in ipsilateral PNs in the RSC of HI mice compared to PNs recorded from the contralateral RSC and the left and right RSCs of sham mice (*p* < 0.001, One-way ANOVA with Tukey’s test; Figure 4B). No significant differences were observed among PNs recorded from the left, right, or contralateral RSC (Figure 4B).

In addition to bursting activity, clinical HIE patients have exhibited changes in EEG coherence across brain regions, which have been correlated with impaired brain functions.^12^ Therefore, we further analyzed neuronal coherence between PNs in the RSC across experimental groups and found that coherence in the ipsilateral RSC was significantly elevated (*p* < 0.01 for ipsilateral vs. contralateral; *p* < 0.001 for ipsilateral vs. sham left and right; One-way ANOVA with Tukey’s test), indicating increased firing synchrony among PNs in the ipsilateral RSC (Figure 4C). Representative cross-correlograms of cell pairs from the RSC further supported this finding, showing that PNs in the sham and contralateral RSC exhibited weakly correlated spike timing, whereas the ipsilateral RSC of HI mice exhibited strong spike-time synchronization between cell pairs (Figure 4D). Additional examples of RSC PN cross-correlograms are shown in Figure S4A, with a similar demonstration of enhanced synchrony in the HI ipsilateral RSC.

To further investigate the contribution of altered firing patterns of PNs induced by HI insult to local field potentials (LFPs) in the RSC, we analyzed the power spectra of LFPs in the left and right RSC of sham mice, as well as the contralateral and ipsilateral RSC of HI mice (Figure S5A). While the sham groups exhibited neuronal activities across all frequency bands, both the contralateral and ipsilateral RSC of HI mice showed a slight increase in power within the delta band (0–4 Hz). Notably, the LFP in the ipsilateral RSC displayed a distinct oscillation, particularly in the theta band (4–8 Hz; indicated by a red arrow), compared to the left and right RSC of sham mice and the contralateral RSC of HI mice.

Analyses of individual animals were conducted to further validate this finding (Figures S6A–S6D). A strong oscillation in the theta band (4–8 Hz) was consistently observed in the ipsilateral RSC of each HI animal. To quantify this observation, the area under the curve for each oscillation band was calculated (Figure S6E). Although statistical significance was not reached, increased LFP activity in the theta band (4–8 Hz) was consistently observed in the ipsilateral RSC. Although developmental changes in oscillatory frequency characteristics, especially in the delta band, have been documented.^43^ Our recordings were conducted in adult mice, where delta, theta, and alpha frequencies exhibit stable characteristics and reflect stable physiological patterns, allowing for valid comparisons across experimental groups.

**Figure 5:**
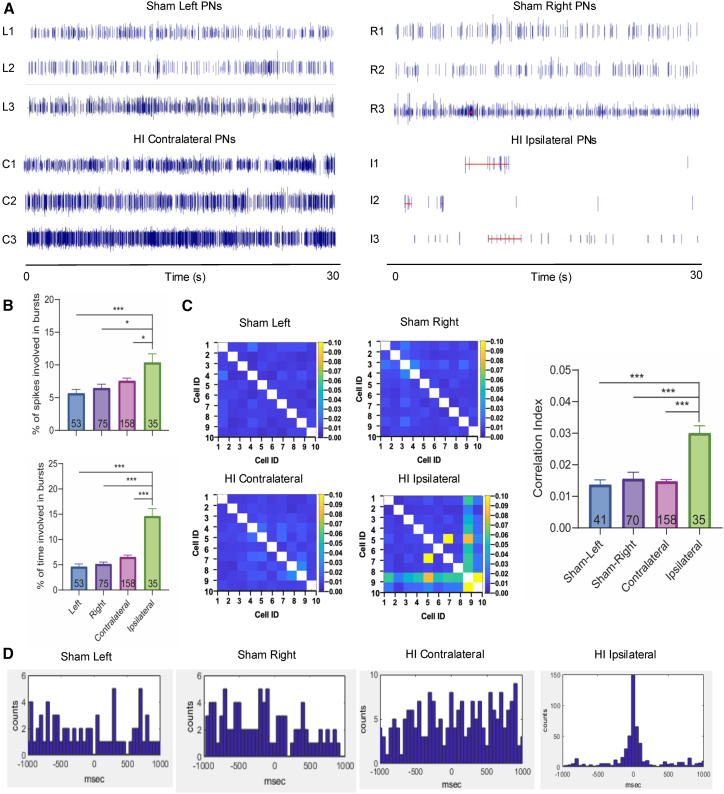
Neonatal HI impairs the firing rate and patterns of hippocampus neurons (A) Representative examples of raster plots showing the neuronal discharge patterns of hippocampus PNs from the right and left hemisphere of sham mice, and the contralateral and ipsilateral hemispheres of HI mice. Random discharge patterns of hippocampus PNs were found in sham mice and the HI contralateral hemisphere. The firing pattern of the hippocampal PNs in the HI ipsilateral hemisphere was characterized by increased burst discharges and synchronization. Neuronal bursts are highlighted with red lines. (B) The analyses of the % of total number of spikes in burst discharge (left) and the % of time spent in burst discharge (right) were significantly (*p* < 0.05 for ipsilateral vs. contralateral and sham right in the left graph; *p* < 0.001 for all other comparisons) increased by HI insult. (C) Ipsilateral PNs in cross-correlation diagrams showed significant (*p* < 0.001) increases in coherence that suggested synchronized neuronal activities. (D) Cross-correlograms of cell pairs in the hippocampus suggested that cell pairs from sham and HI contralateral hemispheres had weakly correlated spike times, leading to dispersive spike time peaks. The HI ipsilateral hemisphere, however, exhibited strong synchronization between neuronal pairs with a sharp peak centered at zero msec. (∗, *p* < 0.05, ∗∗∗, *p* < 0.001; One-way ANOVA with Tukey’s test) See also Figure S7.

Similar to the findings in the RSC, hippocampal PNs also exhibited altered firing patterns, with significantly increased bursting activities as shown by raster plots (Figure 5A) and by the percentage of time and spikes involved in bursting (Figure 5B). Specifically, the percentage of time spent in burst discharges was significantly higher in the ipsilateral hippocampus compared to the contralateral hemisphere and the right hippocampus of sham mice (*p* < 0.05), and all other comparisons also showed significance (*p* < 0.001, One-way ANOVA with Tukey’s test). In addition, neuronal coherence was significantly increased (*p* < 0.001, One-way ANOVA with Tukey’s test; Figure 5C), and cross-correlograms of cell pairs revealed increased spike-time synchronization in the ipsilateral hippocampus (Figure 5D). Additional examples of hippocampal PN cross-correlograms are shown in Figure S4B. Consistently, LFP analysis in the hippocampal revealed pathological oscillations in the ipsilateral hemisphere of HI mice (Figure S5B). Notably, theta (4–8 Hz) and alpha (8–12 Hz) band activities in the ipsilateral hippocampus were significantly elevated (*p* < 0.05, One-way ANOVA with Tukey’s test), whereas beta band (12–30 Hz) activity was significantly reduced (*p* < 0.01 for sham right vs. HI ipsilateral), along with reductions in gamma band (30–50 Hz) (*p* < 0.05 for sham left vs. HI ipsilateral and contralateral; *p* < 0.01 for sham right vs. HI ipsilateral and contralateral, One-way ANOVA with Tukey’s test; Figure S7E), compared to the left and/or right hippocampus in sham mice and/or the contralateral hippocampus in HI mice.

This pattern was further confirmed in analyses of individual animals (Figures S7A–S7D), which consistently showed increased theta-to-alpha oscillations and decreased beta-to-gamma oscillations in the ipsilateral hemisphere of hippocampus of HI mice.

### Neonatal HI results in pathological oscillations and synchrony of pyramidal neuron activities

**Figure 6:**
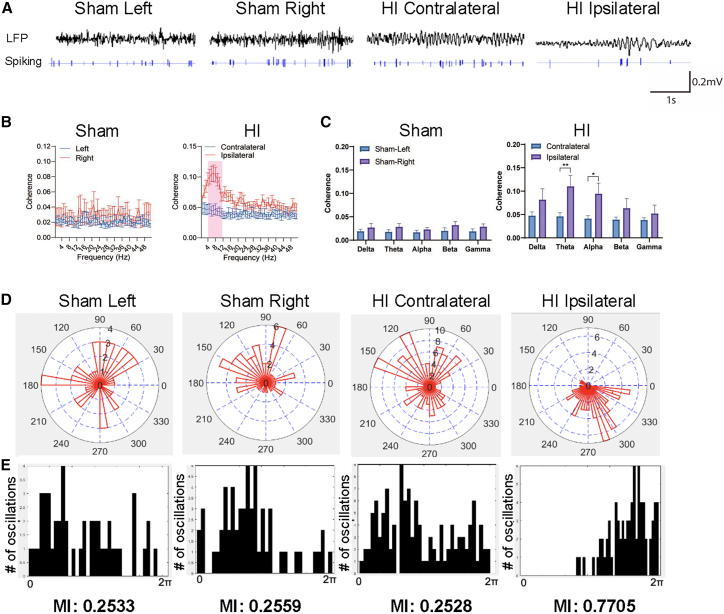
Neonatal HI triggered pathological spike field coherence in the RSC (A) Representative LFP and corresponding spike trains from an RSC PN recorded simultaneously from the left (1) and right (2) hemisphere of a sham mouse, and contralateral (3) and ipsilateral (4) hemisphere of an HI mouse. (B) Analysis of the spike-field coherence in the 1–50 Hz range exhibited a prominent peak in the theta-to-alpha band (1–12 Hz). (C) The ipsilateral hemisphere exhibited significantly (*p* < 0.05 and *p* < 0.01 for alpha and theta band, respectively) increased coherence in the theta and alpha band compared to the contralateral hemisphere. Comparison of spike-LFP activities suggested that neurons involved in low frequency theta-to-alpha oscillations. (D) Results of polar-histograms (upper) and phase histograms (lower) of the spike-field coherence phase distribution in theta-to-alpha band (4–12 Hz) of the RSC. Phase-locking bias between spikes and LFP was evident in the injured ipsilateral hemisphere of the brain, which was absent in both the sham mouse and contralateral side of HI-treated mice. Ipsilateral hemisphere showed a high modulation index (MI) compared with sham and contralateral hemispheres meaning that the theta-to-alpha band exhibited phase-locking bias between spikes and LFP. The phase locking value (PLV) was converted to degree through: PLV value (−1) to 1 = a, Sin (a) = degrees. (E) Results of polar-histogram with points on the unit circle (upper) and angular histograms (lower) of the spike-field coherence phase distribution in theta-to-alpha band (4–12 Hz) with the red line representing the mean resultant vector length for each hemisphere. Phase-locking bias between spikes and LFP was evident and statistically significant by Rayleigh Test in the RSC of the injured side of the brain, which was absent in both the sham mouse and contralateral side of HI mice. (∗, *p* < 0.05, ∗∗, *p* < 0.01; two-way ANOVA with Sidak’s multiple comparison test) see also Figure S8.

Thus far, we have separately identified changes in firing patterns of single-unit PNs and in LFPs induced by neonatal HI brain injury. In addition to these individual effects, we were also interested in changes in the relationship between individual neuronal spikes and LFPs in the RSC and hippocampus following neonatal HI brain injury, aiming to determine whether abnormal neuronal bursts contribute to pathological oscillations in LFP. Representative LFP traces and corresponding raster plots containing spike trains of single PN recorded from the left and right RSC of a sham mouse, and the contralateral and ipsilateral RSC of an HI mouse, are shown in Figure 6A.

Spike-field coherence was calculated between each recorded spike train and the corresponding LFP from RSC PNs in all four experimental groups. Analysis of spike-field coherence across all recorded signals revealed that PNs from the ipsilateral RSC exhibited markedly higher spike-field coherence across all frequencies, with a specific increase in the low-frequency range (1–12 Hz; highlighted in pink), indicating a putative delta-to-alpha (0–12 Hz) oscillation in the RSC (Figure 6B). The spike-field coherences values were then grouped into standard frequency bands: delta (0–4 Hz), theta (4–8 Hz), alpha (8–12 Hz), beta (12–30 Hz), and gamma (30–50 Hz) to further confirm this finding using significance testing. With two-way ANOVA with Sidak’s multiple comparison, we found that the spike-field coherence of ipsilateral PNs and LFPs in the RSC was significantly elevated in the theta (*p* < 0.01) and alpha (*p* < 0.05) bands (Figure 6C).

The same spike-field coherence analysis was conducted for individual sham and HI animals and revealed similar pathological low-frequency oscillation in the ipsilateral RSC of HI mice (Figures S8A and S8B). Based on these findings, we further examined the theta-to-alpha band (4–12 Hz), which exhibited the strongest and most significant pathological oscillation. Our goal was to identify the key contributor to the abnormal spike-field coherence in this frequency range.

Phase synchronization, which reflects the dynamic phase relationship between two oscillatory neural sources,^44^ was examined in the 4–12 Hz range using two visualization methods: polar histograms (Figure 6D, upper) and phase histograms (Figure 6D, lower). The polar histograms display oscillation phase across 0–360°, with concentric circles indicating the number of oscillations at a given phase (i.e., the number of oscillations occurring at the same time). Similarly, the phase histograms present the same data over 0-2π radians. In the sham mice (left and right RSC) and the contralateral RSC of HI mice, phase distributions were random and unbiased. In contrast, the ipsilateral RSC of HI mice showed a clear phase-locking bias at a specific angle in the theta-to-alpha band.

Further illustration of phase distribution is provided in Figure S8C, showing individual phase-locking values (PLVs) and a resultant vector (red line) for each experimental group, indicating the direction and strength of phase concentration. The ipsilateral RSC exhibited a tightly clustered PLVs distribution and the longest resultant vector pointing to a specific phase angle, confirming strong phase-locking bias not observed in other groups.

To quantify phase-amplitude coupling, we calculated the modulation index (MI), which ranges from 0 (no coupling) to 1 (strong coupling).^45^ MIs were computed for each experimental group (Figure 6D). The theta-to-alpha band oscillation in the ipsilateral RSC showed a high MI (MI = 0.7705) compared with left (MI = 0.2533) and right (MI = 0.2559) RSC of sham mice and the contralateral (MI = 0.2528) RSC of the HI mice. These findings further supported the conclusion that phase-locking coupling between spikes and LFPs in the 4–12 Hz range is a pathological feature of the ipsilateral RSC following neonatal HI brain injury.

**Figure 7:**
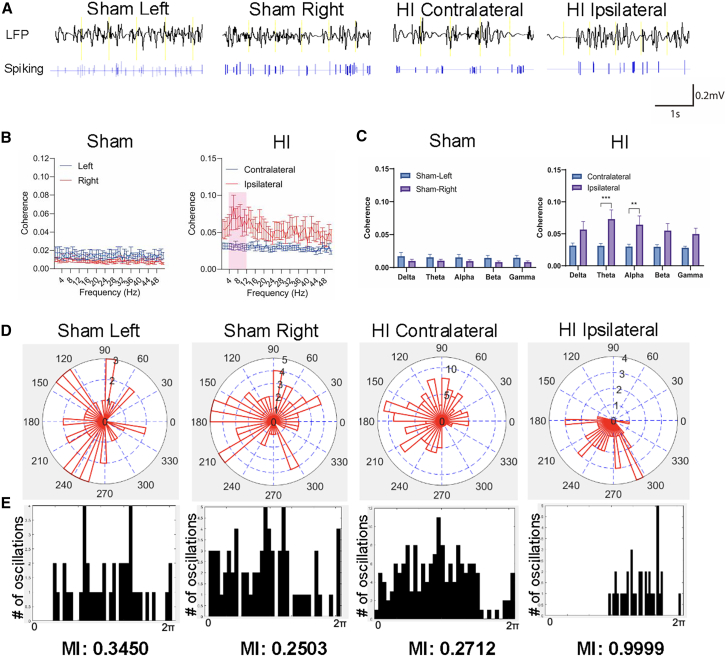
Neonatal HI triggered pathological spike field coherence in the hippocampus (A) Representative LFP and corresponding spike trains from a hippocampal PN recorded simultaneously from the left (1) and right (2) hemisphere of a sham mouse, and contralateral (A.3) and ipsilateral (A.4) hemisphere of an HI mouse. (B) Analysis of the spike-field coherence in the 1-50 Hz range exhibited a prominent peak in the theta-to-alpha band (4–12 Hz). (C) The ipsilateral hemisphere exhibited significantly (*p* < 0.01 and *p* < 0.001 for alpha and theta band, respectively) increased coherence in the theta and alpha band compared to the contralateral hemisphere. Comparison of spike-LFP activities suggested that neurons involved in low frequency theta-to-alpha oscillations. (D) Results of polar-histogram (upper) and phase histograms (lower) of the spike-field coherence phase distribution in theta-to-alpha band (4–12 Hz) of the hippocampus. Phase-locking bias between spikes and LFP was evident in the injured ipsilateral hemisphere, which was absent in both the sham mouse and contralateral side of HI mice. The ipsilateral hemisphere showed a high modulation index (MI) compared with sham and contralateral hemispheres, meaning that the theta band exhibited phase-locking bias between spikes and LFP on the ipsilateral side. The phase locking value (PLV) was converted to degree through: PLV value (−1) to 1 = a, Sin (a) = degrees. (E) Results of polar-histogram with points on the unit circle (upper) and angular histograms (lower) of the spike-field coherence phase distribution in theta-to-alpha band (4–12 Hz) with the red line representing the mean resultant vector length for each hemisphere. Phase-locking bias between spikes and LFP was evident and statistically significant by Rayleigh test in the hippocampus of the injured side of the brain, which was absent in both the sham mouse and contralateral side of HI mice. (∗∗, *p* < 0.01, ∗∗∗, *p* < 0.001; two-way ANOVA with Sidak’s multiple comparison test) See also Figure S9.

The same set of analyses was performed on recordings of PNs from the hippocampus of sham and HI mice. Representative comparisons between spike trains and their corresponding LFPs from hippocampal PNs in each experimental group are shown in Figure 7A. Spike-field coherence between PNs and LFPs in the ipsilateral hippocampus again showed increases across all frequency ranges, with a particularly notable increase in the low-frequency bands (1–12 Hz) highlighted in pink (Figure 7B). When spike-field coherences were grouped by frequency bands, significant increases were observed in the theta (*p* < 0.001) and alpha (*p* < 0.01) bands compares to those of the contralateral hippocampus (Figure 7C).

Analyses of individual animals further confirmed that PNs and LFP from the ipsilateral hippocampus of HI mice exhibited elevated spike-field coherence across all frequency bands, with the most predominant increases in the theta and alpha bands (Figures S9A and S9B), suggesting a pathological theta-to-alpha oscillation. Phase synchronizations at these frequency bands were examined in more detail and are presented as polar histograms (Figure 7D, upper) and phase histograms (Figure 7D, lower). The theta-to-alpha oscillations in the left and right hippocampus of sham mice and the contralateral hippocampus of HI mice showed randomly distributed phases. In contrast, the ipsilateral hippocampus of HI mice displayed phase distributions clustered around a specific angle, suggesting a phase-locking bias.

An alternative illustration of the phase distribution in Figure S9C confirmed that the ipsilateral hippocampus exhibited a concentrated distribution of PLVs across 0–360°, along with the longest resultant vector pointing to a specific angle, indicating strong phase-locking bias not observed in the left and right hippocampus of sham mice or the contralateral hippocampus of HI mice. Results from MI analysis further confirmed this finding, that is the ipsilateral hippocampus (MI = 0.9999) showed higher phase-amplitude coupling compared to the left (MI = 0.3450) and right (MI = 0.2503) hippocampus of sham mice and the contralateral hippocampus (MI = 0.2712) of HI mice. These results provided additional evidence of a phase-locking bias in theta-to-alpha oscillation between PN spikes and corresponding LFPs in the ipsilateral hippocampus.

### Neonatal HI brain injury reduces cross-correlation in hippocampal pyramidal neurons during motor learning

**Figure 8:**
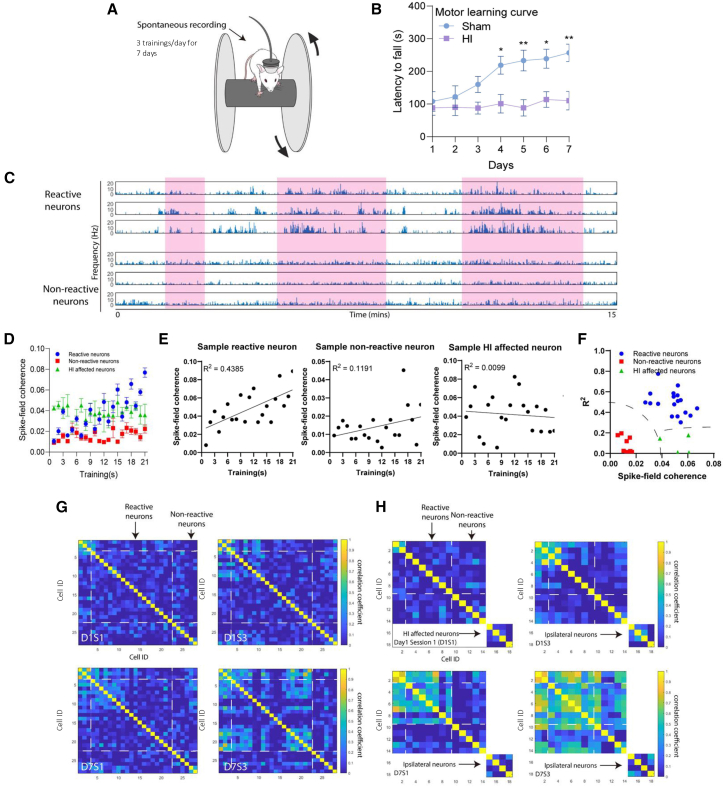
Neuronal dynamics in the hippocampus during rotarod motor learning training sessions (A) A graphic representation of the rotarod apparatus with the tested mouse undergoing simultaneous electrophysiological recordings. (B) Average latency to fall in sham and HI mice on the rotarod across a 7-day testing period (3 trials/day). Rotating drum moved at a constant speed of 10 revolutions per minute. Sham animals showed improvement on days 4–7. (∗, *p* < 0.05, ∗∗, *p* < 0.01, compared to day 1. Two-way ANOVA with Sidak’s multiple comparison test), while HI mice showed no difference in performance across the 7 days. (C) The spike-field coherence of PNs involved in motor learning across 21 training sessions over 7 days. HI-affected neurons showed a decreasing trend in coherence compared to reactive neurons. (D) Raster plots showing 3 physiological types of cells exhibiting different firing patterns across training sessions and classified as (1) constant high activity, (2) increased activity across sessions (reactive neurons), and (3) constant low activity (non-reactive neurons). (E) Linear regression of neuronal activities across 7 days were evaluated and classified into 2 physiological types (reactive and non-reactive) and HI-affected. Classification of recorded hippocampal PNs during the 7-day training using regression coefficient (R2) (Li et al., 2017). Non-reactive neurons exhibited low R2 and low spike field coherence, while reactive neurons exhibited high R2 and high spike field coherence. HI-affected neurons showed pathological characteristics with low R2 and high spike field coherence. (F) Regression coefficients (R^2^ values) showed HI-affected PNs did not align with either reactive or non-reactive categories. (G) Pairwise cross-correlation matrix of 28 hippocampal PNs recorded from one representative sham mouse. PNs are ordered (Cell #ID) according to the sequence of hierarchical clustering. Cross-correlation between reactive neurons was increased after 7 days of training while non-reactive neurons remained low. (H) Pairwise cross-correlation matrix of 18 hippocampal PNs recorded from one representative HI mouse. The squares from top to bottom segregate reactive neurons, non-reactive neurons and HI-affected neurons identified. Increased cross-correlations are evident among reactive neurons in the contralateral hemisphere after training. HI-affected neurons in the ipsilateral hemisphere showed constant low cross-correlation throughout training sessions. See also Figure S10.

A group of hippocampal neurons was traced throughout a 7-day rotarod motor learning protocol to investigate the impact of neonatal HI brain injury on neuronal activity and dynamics during neurobehavior. As shown in Figure 8A, *in vivo* electrophysiological recordings were performed to record neuronal activity throughout the training sessions. Mice were trained to run on the rotarod at a constant speed of 10 revolutions per minute for up to 300 s^46^ Three trials per day were conducted over seven consecutive days, with each trial ending either when the mouse fell or when the time limit was reached. While sham mice showed significant increases in latency to fall from day 4 to day 7 (*p* < 0.05 or *p* < 0.01 compared to day 1, unpaired *t* test), indicating normal motor skill learning. In contrast, HI mice showed no improvement across the 7-day training period, suggesting a deficit in motor skill acquisition (Figure 8B).

In sham mice recorded during rotarod training, 28 hippocampal PNs were traced across 21 training sessions over 7 days. Figure S10 shows an example of three neurons traced over 7 days, displaying similar or identical waveforms and consistent clustering on energy plots over time. These 28 traced hippocampal PNs were further classified into two types based on activity during training: (1) neurons that increased their activity after training (“reactive neurons”) and (2) neurons with consistent low activity throughout sessions (“non-reactive neurons”). Representative raster plots of three reactive and three non-reactive neurons from a single training day are shown in Figure 8C, with the three training sessions highlighted in pink.

To characterize changes in hippocampal PN firing patterns in sham and HI mice, spike-field coherence was measured between PN spikes and corresponding LFPs. We found that spike-field coherences in HI-affected PNs from the ipsilateral hippocampus across 21 training sessions differed from those of both reactive and non-reactive neurons in the sham mice (Figure 8D). Regression analyses were conducted across three groups of PNs (reactive, non-reactive, and HI-affected) to assess the relationship between spike-field coherence (real-time neuronal and LFP activities) and training status (rotarod on/off) (Figure 8E). Regression coefficients (R^2^ values) indicated that HI-affected PNs did not align with either reactive or non-reactive categories (Figure 8F). Specifically, HI-affected PNs showed higher coherence than non-reactive neurons but lower R^2^ values than reactive neurons. This suggests that HI-affected PNs exhibited pathological characteristics potentially contributing to motor learning deficits following HI brain injury.

Furthermore, to assess the dynamic changes in PN activity during rotarod training, neuronal correlation coefficient matrices were generated for each group (reactive, non-reactive, and HI-affected) across all training sessions. Cross-correlations were compared between short-term sessions within the same day (e.g., day 1 session 1 vs. session 3) and long-term sessions across different days (e.g., day 1 session 3 vs. day 7 session 3). Reactive neurons in sham mice showed increased cross-correlations with both short- and long-term training (Figure 8G). In HI mice, 18 hippocampal PNs were traced from both hemispheres and classified as reactive or non-reactive if from the contralateral side, or as HI-affected if from the ipsilateral hippocampus. Correlation coefficient matrices showed that HI-affected PNs remained in a relatively low activity state throughout 21 sessions, in contrast to reactive neurons in the contralateral hippocampus, which showed enhanced activity after training (Figure 8H).

In summary, our findings linked motor learning deficits following HI brain injury to pathological neuronal activity in the hippocampus. Specifically, PNs in the ipsilateral hippocampus exhibited reduced cross-correlations during both short- and long-term training sessions and resembled characteristics of non-reactive neurons, but not reactive neurons essential for normal motor learning observed in the sham and contralateral hippocampus. These results provided neuronal-level evidence of altered PN activity underlying motor learning impairments following HI brain injury.

## Discussion

In this study, we conducted simultaneous *in vivo* electrophysiological recordings in the RSC and hippocampus of adult mice to evaluate the long-term neurodevelopmental impact of HI brain injury at neonatal age. We observed abnormal bursting and synchrony patterns in surviving PNs of the ipsilateral RSC and hippocampus, which differed from sham mice and were considered pathological. These PNs exhibited spike-field coherence phase-locked to the theta-to-alpha oscillatory band. Additionally, mice subjected to neonatal HI displayed impaired motor learning behavior, coinciding with aberrant PN firing patterns, providing the first evidence linking disrupted RSC-hippocampus neuronal communications to long-term motor learning deficits following neonatal HI.

The RSC and hippocampus interact bidirectionally with motor and thalamic regions, showing increased theta coherence during learning to integrate spatial context into motor control, forming a critical network that supports sensorimotor and spatial learning.^47^ Studies have shown that perinatal HIE leads to both acute and long-term impairments in movement, learning, and memory,^30^ affecting over 50% of HIE-affected infants, with deficits often undetected until school age.^48^^,^^49^ However, the impact of neonatal HI on the developmental process and maturation of the RSC-hippocampus circuit remains poorly understood. In this study, our recordings demonstrated that neonatal HI led to persistent alterations in neuronal activity in the RSC and hippocampus in adulthood, coinciding with impaired motor learning. Functionally, these two regions are part of a broader circuit that includes motor, entorhinal, and thalamic structures. Their dynamic interaction and coordination are essential for translating spatial memory and contextual cues into motor output.^50^ Notably, coherence between the RSC and hippocampus, particularly in theta and alpha frequency bands, increases during motor skill learning behavior. In line with this, our findings showed synchronized neuronal activity and pathological oscillations in ipsilateral PNs following HI brain injury, suggesting long-term disruptions in functional connectivity within the RSC-hippocampal circuit.

While the Vannucci model remains an animal model for investigating the mechanisms of neonatal hypoxic brain injury, this HIBI model has been widely accepted in animal studies to investigate the potential mechanisms underlying HI-mediated brain injury in the neonates, which may lead to HIE and/or cerebral palsy in the long term.^51^ With this well-established model, we observed about 40% evident morphological brain damage in long-term survivors. These structural changes were accompanied by impaired short-term neurobehavior, indicating that neonatal HI disrupts brain development, and such impairment persisted to adulthood, affecting both motor function and motor learning. In line with previous findings,^25^^,^^26^ we recorded significantly fewer neurons in HI-affected brain regions and found that PNs, but not INs, were particularly vulnerable, likely due to their lower glutathione levels.^52^ Disrupted PN activity likely impairs RSC-hippocampal coordination, contributing to learning and memory dysfunctions.

Although early studies have reported EEG and LFP abnormalities post-HI,^6^ few have examined *in vivo* neuronal firing patterns in the RSC and hippocampus at the single-cell level. Our work addressed this gap by recording and analyzing both single-unit and population-level activities during memory-related behavioral tasks that involve the RSC and hippocampal formation.^53^^,^^54^^,^^55^ We found increased PN bursting and pathological LFP oscillations, mirroring EEG abnormalities in HIE patients.^12^^,^^22^ Such local and large-scale hyper-synchrony depends on HIE severity^11^ and disrupts network function.

We further explored the mechanisms underlying the identified pathological firing patterns and their effects on the motor circuit. While frequency band features can shift across development, our recordings were performed in adult mice, where cortical and hippocampal delta, theta, and alpha rhythms are reliably established.^56^ As a transmitting terminal of the memory network, RSC-hippocampal dysfunction may underlie behavior deficits in the adult who survived from neonatal HIE. In particular, exaggerated hippocampal theta oscillations may explain the impaired motor learning observed in HI mice. This aligns with previous studies linking disrupted theta oscillation in cingulate cortices (including the RSC) to memory impairments.^57^ Similarly, in Parkinson’s disease, motor impairments are associated with abnormal beta-bursts and excessive spike-FLP coupling.^58^ Our findings showed heightened spike-field coherence across delta, theta, and alpha bands in the ipsilateral hemisphere, accompanied by phase-locking bias, suggesting pathological over-synchronization.

Motor learning acquisition involves neurotransmission, as seen in rotarod tests.^59^ Even without real-time neuronal spiking profiles, the organization of functional neuronal clusters in layer 2/3 of the motor cortex can predict motor behavior.^60^ However, the direct contribution of PN activity to motor learning has not been characterized. In our rotarod experiments, we identified “reactive” and “non-reactive” neurons in sham mice. Reactive PNs exhibited increased activity and pairwise correlations over time, while ipsilateral PNs in the HI mice showed persistently low activity and low pairwise correlations. These altered dynamics suggest that HI impairs the ability of hippocampal PN adaptation during motor learning.

In conclusion, our study reveals that neonatal HI brain injury leads to long-term disruption in PN activity and network synchronization in the RSC and hippocampus. These changes impair motor learning and provide neuronal mechanistic insight into the neurobehavioral deficits observed in clinical HIE survivors. Our findings highlighted the potential strategies targeting the dynamics of the RSC-hippocampal network in developing new therapies for long-term HIE recovery.

## Resource availability

### Lead contact

Further information and requests for resources and reagents should be directed to and will be fulfilled by the lead contact, Zhong-Ping Feng (zp.feng@utoronto.ca).

### Materials availability

This study did not generate new unique reagents. All commercially available materials used in this study are listed in the key resources table.

### Data and code availability

All MATLAB analysis scripts and data supporting the findings of this study are available at https://doi.org/10.5281/zenodo.18451609.

## Acknowledgments

This work was supported by the 10.13039/501100000024Canadian Institutes of Health Research (CIHR-PJT-153155 and PJT-191824) awarded to Z.-P.F. M.Y., and A.O. were the recipients of the Ontario Graduate Scholarship (Doctoral).

## Author contributions

L.D. and M.Y. contributed to data acquisition, analysis, and interpretation. H.W.S. performed the tetrode implantation surgeries. H.W.S. performed the tetrode implantation surgeries and wrote matlab analysis code. R.H. conducted the behavioral testing. S.S., K.T.-N., L.M., and Z.-P.F. provided insights on data interpretation. L.D. and M.Y. drafted the original manuscript. L.D., M.Y., Z.L., A.O., and Z.-P.F. revised manuscript. Z.-P.F. conceptualized the study, designed the experiments, and supervised the overall project.

## Declaration of interests

The authors declare no competing interests.

## Declaration of generative AI and AI-assisted technologies in the writing process

During the preparation of this manuscript, the authors used ChatGPT to assist with grammar checking. After using this tool/service, the authors reviewed and edited the content as needed and take full responsibility for the content of the publication.

## STAR★Methods

### Key resources table

**Table:** 

REAGENT or RESOURCE	SOURCE	IDENTIFIER
**Chemicals, peptides, and recombinant proteins**		

2,3,5-triphenyltetrazolium chloride	Sigma Aldrich	T8877
Paraformaldehyde (PFA)	BioShop	PAR070
Normal Goat Serum	Gibco	16210064
4′,6-diamidino-2-phenylindole (DAPI)	Roche	10236276001
Phosphate-Buffered Saline (PBS)	Gibco	10010023

**Experimental models: Organisms/strains**		

CD-1 mouse	Charles River Laboratories	Strain Code 022; used at P7, both sexes

**Software and algorithms**		

MATLAB	MathWorks	R2019b https://www.mathworks.com
Customized MATLAB scripts	This paper	https://doi.org/10.5281/zenodo.17195940
ImageJ	NIH	https://imagej.net/ij/
Prism GraphPad	GraphPad Software	https://www.graphpad.com/

### Experimental model and study participant details

#### Animals

CD1 mice were purchased from Charles River Laboratories (Sherbrooke, QC, Canada) and maintained at the animal facility of the University of Toronto. All animal procedures (AUP#20012378; #20011560) complied with the Canadian Council on Animal Care (CCAC) guidelines and were approved by the University of Toronto Animal Care Committee (Office of Research Ethics). Mice were housed under standard conditions (12 h light/dark cycle, lights on 7:00 am–7:00 pm) with *ad libitum* access to food and water.

Both male and female pups were used at postnatal day 7 (P7) and randomly assigned to sham or hypoxic-ischemic (HI) groups. For histological validation, 40 HI mice were used for TTC staining at P8, and 18 HI mice at 4 months of age. *In vivo* electrode arrays were implanted in two sham and three HI mice for electrophysiological recordings. Six sham and six HI mice were used for long-term motor learning tests.

#### Sex as a biological variable

Both sexes were included in this study. Data were not analyzed by sex because of limited sample size, which is acknowledged as a limitation.

### Method details

#### Neonatal hypoxic-ischemic brain injury

The Rice-Vannucci model of hypoxic-ischemic brain injury in the neonatal mice^51^^,^^61^ was used in this study, as described previously.^35^^,^^36^^,^^37^^,^^62^^,^^63^ Postnatal day 7 (P7) mouse pups of either sex were divided into either the sham control group or the HI-induced group. P7 mice were anesthetized with isoflurane (3.0% for induction and 1.5% for maintenance) and maintained with a 37°C heat pad. The right common carotid artery was isolated and ligated with a bipolar electrocoagulation device (Vetroson V-10 Bipolar electrosurgical unit, Summit Hill Laboratories, Tinton Falls, NJ, USA). Pups were then returned to their home cage with 37 °C heat support and allowed to recover for 1.5 hours. After the recovery period, hypoxia was induced by placing the pups in a 37 °C airtight hypoxic chamber (A- Chamber A-15274 with ProOx 110 Oxygen Controller, Biospherix, NY, USA). The chamber was perfused with a gas mixture of 7.5% oxygen and 92.5% nitrogen for 60 minutes. The in-chamber temperature and oxygen level were continuously monitored by a homoeothermic blanket control unit (K-017484 Harvard Apparatus, Massachusetts, USA) and an oxygen sensor (E-720 Sensor, Biospherix, NY, USA). All pups were returned to their home cage and held under standard conditions during recovery. Sham animals were anesthetized and underwent artery exposure without ligation and hypoxia procedures.

#### TTC staining/infarct volume measurement

To test the validity of HI modeling, 40% of HI-treated mice were randomly selected for 2,3,5-triphenyltetrazolium chloride (TTC) staining to visualize and examine the brain infarction volume. Brains were extracted and coronally sectioned into four ∼1 mm slices at 24 hours post-HI. Brain slices were stained with 1.5% TTC for 20 minutes at 37 °C to visualize infarcted areas. All images were analyzed by ImageJ (NIH, USA), and infarcted regions were quantified as follows: Corrected infarct volume (CIV), (%) = [contralateral hemisphere volume − (ipsilateral hemisphere volume − infarct volume)] / contralateral hemisphere volume × 100 (Sun et al. 2014, Chen et al., 2015).

#### Immunofluorescence staining

Animals were perfused under deep anesthesia with normal saline and 4% paraformaldehyde solution (PFA). Once the brains were retrieved, they were fixed in 4% PFA solution. Brains were retrieved and mounted in cryo-embedding media before proceeding to cryo-sectioning. Coronal sections (30μm) were obtained with a freezing microtome (Leica, Germany). All free-floating sections were blocked with 2% normal goat serum (NGS) for 1 hour at room temperature. After blocking, all floating sections were washed with phosphate-buffered saline (PBS) and then incubated with 4′,6-diamidino-2-phenylindole (DAPI) for 20 minutes. After incubation, all floating sections were washed with PBS and proceeded to mounting. All sections were mounted onto Superfrost slides and cover-slipped for Zeiss LSM 700 Confocal Microscope imaging. Images were processed in black and white for the representation of brain morphology and the location of tetrodes.

#### Short-term behavioral assessments

General procedures of short-term behavioral tests are reported in our previous works,^62^^,^^63^ including (1) the geotaxic reflex, (2) cliff aversion reflex, and (3) grip test, were performed to assess HI recovery on 1, 3, and 7 days after surgery (i.e., postnatal days P8, P10, P14). Briefly, in the geotaxis reflex test, pups were placed head-down on a 45° incline, and the latency to rotate 180° was recorded to assess vestibular and proprioceptive abilities. The cliff aversion reflex evaluates impulse control by placing pups at the edge of a platform and measuring the latency to withdraw both forepaws from the edge. The grip test examines forelimb strength and endurance by suspending pups above a soft pad with their forepaws gripping a horizontal wire and recording the time until release.

#### Rotarod motor learning test

A rotarod apparatus with automatic timers and falling sensors (Harvard Apparatus, USA) was used. The mouse was placed on a 3 cm diameter drum. Before the training sessions, mice were habituated to stay on a stationary drum for up to 3 minutes. Habituation was repeated every day for 1 minute just before the session. The speed of the 3 cm diameter rod was set to 10 revolutions per minute. The animal was returned to its home cage immediately after falling. Each session consisted of 3 rounds of test trials, and the average latency to fall was recorded. A fall was overlooked when the animal remained on the drum for 300 seconds. To evaluate long-term motor learning, the sessions were repeated for 7 consecutive days.

#### Stereotaxic electrode implantation

The scalp of the mouse was shaved and sterilized with betadine and 100% ethanol. An ophthalmic ointment was applied to protect the eyes of the mice. Mice were anesthetized with 1-4% isoflurane and 90% oxygen delivered through a nose mask and secured to a stereotaxic frame via ear bars (Kopf Co.). Mice were injected with intraoperative meloxicam (0.2mg/kg, SC) as an analgesic. Scalp incisions began when there was no longer a reflex response to a toe pinch. The fascia was bluntly dissected from the skull to expose the cranial sutures. The skull was treated with hydrogen peroxide (3%) to remove blood and dry out the bone. Four small screws were secured to the perimeter of the skull over the parietal and frontal bones on both sides. A ground screw with a lead wire was secured over the cerebellum. A craniotomy (1.5mm X 3.0mm) was made over the hippocampus (with the center positioned at AP -2mm, ML 1.5mm). The long dimension of the craniotomy was oriented coronally to the mouse brain. The craniotomy was cleared of dura mater, and the pia mater was incised. The cranial opening was treated with mineral oil to prevent drying of the cortical surface. The hyperdrive was positioned over the top of the craniotomy with a stainless-steel electrode bundle physically abutting the cortical surface. The hyperdrive was secured in place with cyanoacrylate adhesive and dental cement. The nuchal muscles were exposed through blunt dissection, and two 4.0 silk sutures were sewn into the left and right muscles.^38^ Looped EMG wires were fixed to the muscle with silk thread. The ground wire was connected to the hyperdrive, and the excess skin overlying the neck muscle was sutured. Mice were injected with 0.5mL of lactated Ringer solution and allowed to recover under a heating lamp. Special care was taken to avoid over-penetration of the stimulating electrode into the ventral internal capsule. Once the animals showed signs of normal ambulation, they were returned to individual cages and fed a mashed diet until full recovery. Meloxicam (0.2 mg/kg, SC) was provided daily for three days as an analgesic.

#### Electrophysiological recordings

Recordings were conducted with an Open-Ephysiology acquisition system, which was interfaced with an RHD2132 Intan chip providing 32 channels of recording. The headstage was interfaced with an 8-tetrode custom hyperdrive (HypD-8T-M-LB-NT, Neurotek Innovative Technology, Canada). The hyperdrive was loaded with 8 twisted nichrome tetrodes. Tetrodes were plated with a concentrated gold solution to ∼200KOhms to reduce their impedance and tested on a BAK (IMP2) impedance meter. The drive was grounded to the skull with screws (GND-Scr-NT, Neurotek Innovative Technology, Canada). Each day during recording, the animal was attached to the data acquisition system. Signals from the headstage were routed via a commercial commutator (CMTR-12-24-M-INTAN-NT, Neurotek Innovative Technology, Canada) to the acquisition system to prevent cable twisting. Signals were recorded at 30KHz and stored on a PC in ‘open-ephys’ format for offline analysis. Tetrodes were lowered into the deep layers of the cortex or the hippocampus daily at ∼60-120 microns, and signals were monitored until relatively stable units of sufficient amplitude were recorded. The recordings were typically 30 minutes per session per day, and approximately 10 days of recordings were performed on each mouse, depending on how well the signals were. The location of the tetrode on each recording day and the final depth of the tetrode were recorded, which facilitated future analyses. Spikes were detected with filters set between 600Hz and 6KHz. Spikes were detected and saved based on whether they crossed a threshold (typically 50μV) in a window consisting of 40 samples.

#### Spike sorting

The stimulus artifact removal and single-unit spike-sorting procedures were performed in the MClust bundle and Matlab scripts (Mathworks, USA), using a combination of automatic and manual analysis techniques.^64^

To identify spikes occurring on multiple cells simultaneously from a tetrode, single-unit spike-sorting procedures were performed using the MClust bundle and Matlab scripts (Mathworks, USA). Single-cell spikes exhibit distinct waveform characteristics that form clusters in the high-dimensional feature space between tetrodes.^65^^,^^66^ A semi-automated clustering algorithm of KlustaKwik^67^ was applied to produce initial spike clusters by the K-means clustering algorithm and spike valley seeking. After automated clustering, the clusters from individual firing units were visually verified by comparing the energy plot between tetrodes. Only spikes having similar waveforms in a principal component PC space were considered to originate from a single neuron. Spikes from individual firing units (or neurons) that form isolated clusters that are distinct from each other can be observed in a principal component space, and each clustered spike was considered from a single “firing unit”.

#### Burst detection

Neuronal discharge patterns, including regular, irregular, and burst patterns, were characterized based on their respective distributions of autocorrelation. The regular pattern exhibited a symmetric distribution, the irregular pattern followed a Poisson distribution, and the burst pattern displayed a quasi-normal distribution. Simultaneous burst discharge at different timepoints was quantified by the Legendy-Surprise method.^42^ Burst discharges were further quantified by Poisson surprise, i.e. the negative logarithm of the firing probability in a random spike,^42^ with the Poisson surprise threshold set at 5.

#### Neuronal correlations

Cross-correlation analysis and cross-correlograms were applied and generated to study the synchronization level among cortical and hippocampal neurons.^68^ The discharge patterns of neurons were classified into 3 categories: regular, irregular, and burst discharge. This classification was based on how the spike density aligned with the χ2 goodness-of-fit test that displayed the corresponding Vannucci and Back^51^, Harris et al.^55^ The spike density correlogram was plotted by calculating the number of spikes in each successive interval, i.e. equivalent to the reciprocal mean firing rate. The number of occurrences of spikes within each designated time interval was then counted and plotted as autocorrelation or cross-correlation. The probability distribution of the neuron's discharge density was represented by the cross-correlograms.

#### Field-spike coherence and phase-locking

In the frequency domain, the coherence between each neuron's spikes and the simultaneously recorded LFP was calculated under the frequency band set at 0.5 to 100Hz, with frequency blocks of 256 and a 5% window overlap. To study the phase synchronization between spikes and simultaneously recorded LFP, polar histograms and phase histograms were built by filtering the LFP into the theta-to-alpha band (4-12 Hz), and the phase estimates were derived from coherence analysis using blocks of 256 data points (0.4 Hz resolution). The spike that occurred at the peak or trough of LFP was assigned a phase of t 0° or 180°, respectively. The modulation index (MI) was calculated for each experimental group to quantify the phase-amplitude coupling^45^ (script retrieved and modified from Pierre Mégevand 2023).

#### Longitudinal unit tracking

Four measures were employed to evaluate the consistency of single-unit spike waveforms and firing characteristics to determine whether single-unit clusters corresponded to stable tracking of the same neuron over the training period. (1) The similarity of spike waveform shape was quantified as the maximum time-shifted linear correlation coefficient (Max r) between the averaged spike waveform on a given day and day 1, and the resulting coefficient was Fisher-transformed to improve the normality of the distribution. (2) The normalized trough-to-peak amplitude difference (ΔPamp) was computed as the ratio of spike amplitude change on a given day relative to day 1. (3) To characterize single-unit firing properties, we computed log-scaled interspike interval histograms (ISIH) from 0.5 to 105 ms (in 100 bins) and a log-scaled autocorrelogram (±100 ms in 100 bins). We then calculated the Kullback–Leibler (KL) divergence between pairs of normalized ISIHs or autocorrelograms, which were log-transformed to approximate Gaussian distributions. We assumed units recorded in two continuous sessions (10 min each) were stable and used the percentage of the same unit that failed to satisfy the stability criterion as an estimate of the chance of false negatives. The criteria for sorting quality for all included single units remained consistent over the 7-day training period.

To evaluate the joint neuronal firing pattern during motor learning, we calculated pairwise cross-correlation across all pairs of neurons' spike trains in a given period, i.e., rotarod start and fall-off. Spike trains were binned (12.5 ms) to get the instantaneous firing rates, which were then convolved with a Gaussian filter for smoothing. The squared cross-correlation coefficient (R^2^) between pairs of neurons' firing activity was calculated to generate the correlation matrix for each training session. Neurons were ordered following the ranks from hierarchical clustering. The similarity of correlation matrices across different training days was assessed using the correlation coefficient of off-diagonal elements. To account for the possibility that the emerging correlation merely reflected changes in across-trial variability of forelimb reaching kinematics, we also computed correlations for reactive and non-reactive neurons using activities recorded when the animal was not executing the task.

### Quantification and statistical analysis

Unpaired t-tests were performed on the behavior data from HI-induced mice to compare with the Sham group. Two-way ANOVA with Sidak’s multiple comparison was performed to compare the motor performance of mice from different experimental groups at multiple time points. All behavioral test results were shown as mean ± SEM.

Electrophysiological Statistics are presented as mean ± SEM unless otherwise specified. One-way or two-way ANOVA was applied for each pooled result, as indicated in the figure legends, with Tukey’s post hoc or Sidak’s multiple comparison. No statistical methods were used to pre-determine sample sizes. The sample sizes in this study are, in general, similar to those employed in the field.
