# Supplementary material for: The long-term impact of neonatal hypoxic-ischemic brain injury on neural dynamics in deep-layer retrosplenial cortex and hippocampus in mice
Source: iScience. 2026 Mar 28;29(4):115003. doi: 10.1016/j.isci.2026.115003 (PMC13091419; doi:10.1016/j.isci.2026.115003)
Supplement: Document S1. Figures S1–S10 [file mmc1.pdf]

iScience, Volume *29*

Supplemental information

The long-term impact of neonatal hypoxic-ischemic brain injury on neural dynamics in deep-layer retrosplenial cortex and hippocampus in mice

Lida Du, Meng Yang, Hendrik W. Steenland, Zhengwei Luo, Andrea Ovcjak, Ruiyan Hu, Shuzo Sugita, Kaori Takehara-Nishiuchi, Luka Milosevic, and Zhong-Ping Feng

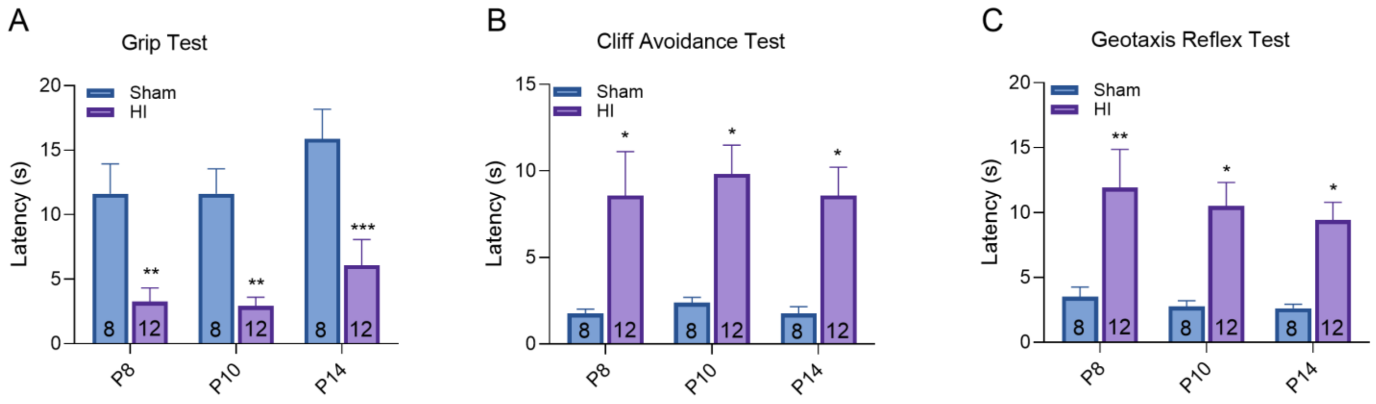

Figure S1 Neonatal HI impaired short-term neurobehavioral outcomes. Short-term behavioral was assessed with grip test (A), cliff avoidance reflex (B), and geotaxis reflex test (C). Shan and HI animals were measured on P8, P10, P14 days (i.e. 1, 3, and 7 days after HI) (*p < 0.05 versus sham, Two-way ANOVA with Sidak’s multiple comparison)

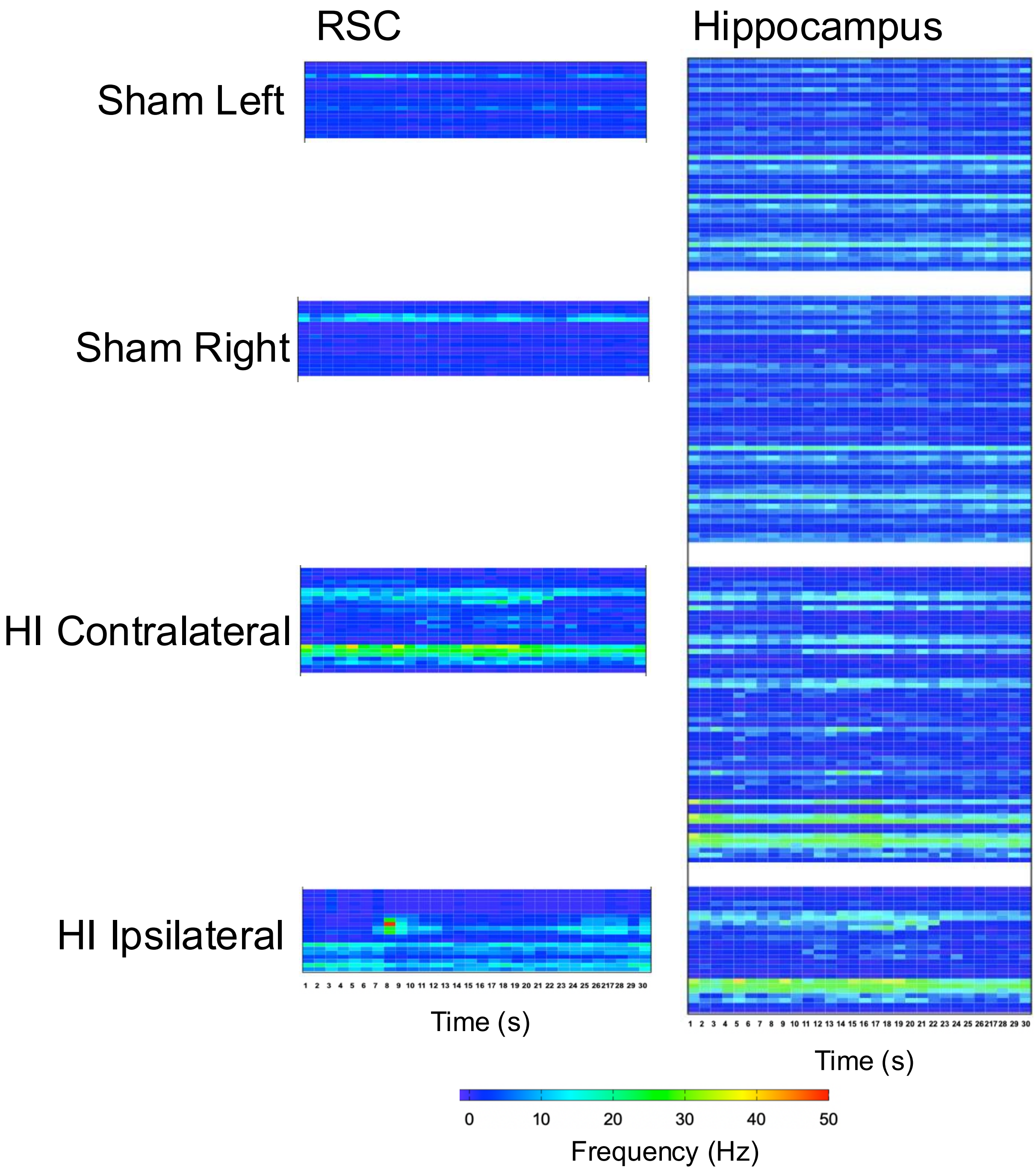

Figure S2 Neonatal HI affects neuronal profiles. Firing frequency of all cells in a sham and a HI mouse during freely moving conditions for a duration of 30 seconds. The contralateral hemisphere in the HI mouse showed complementary activities in the RSC and hippocampus compared with firing frequencies in left and right hemispheres in the sham mouse. Synchronized firing activities were also observed in HI ipsilateral neurons.

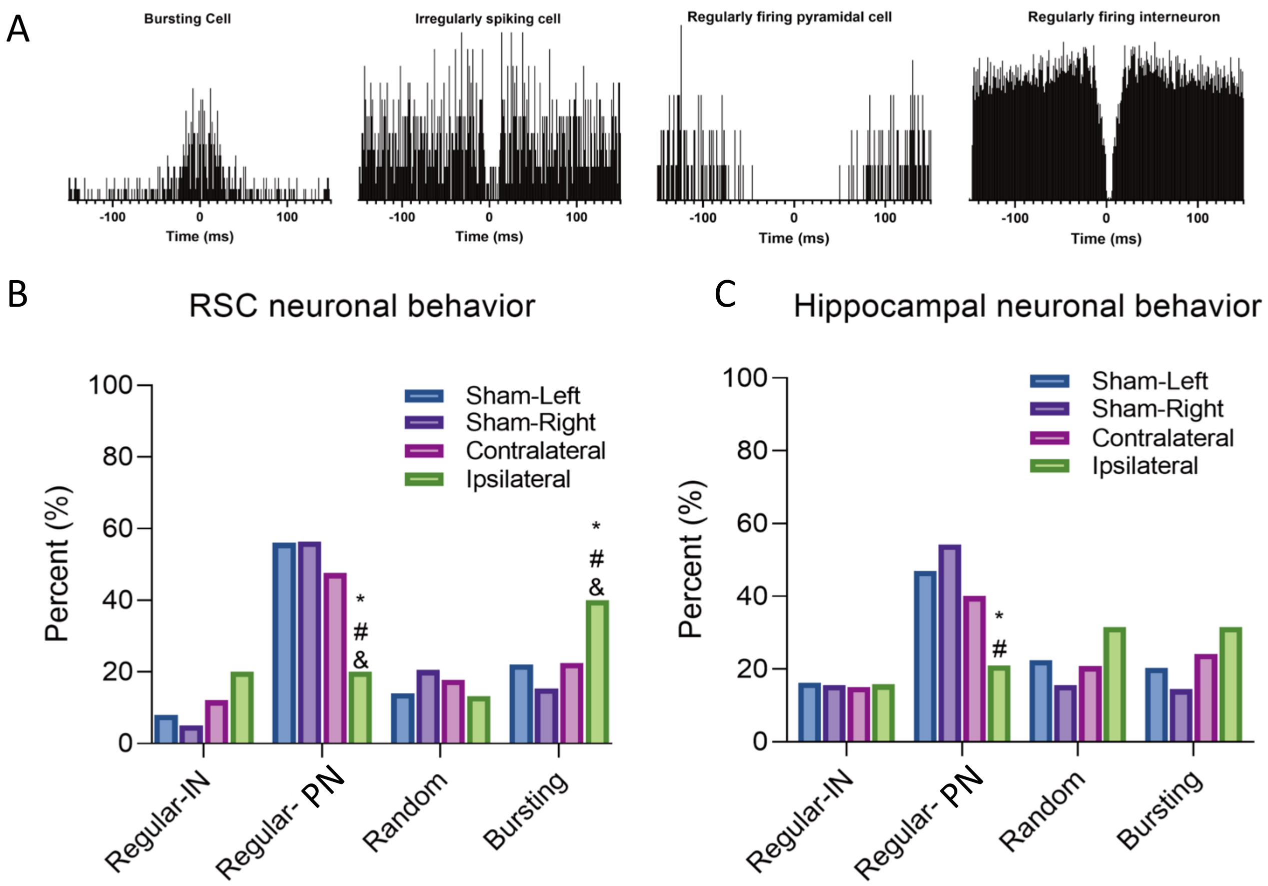

Figure S3 Neonatal HI affects neuronal activities. (A) Auto-correlogram of well isolated spiking units showing neuronal behavior. Putative cells were classified into "bursting", "irregular spiking", "regular spiking" and "spiking interneuron" groups from their auto-correlograms. (B) In the RSC, more ipsilateral neurons tended to burst than contralateral and sham neurons. (C) In the hippocampal CA1, ipsilateral neurons showed a significant increasing tendency ($p = 0.07$) of bursts. Significance between each group was compared by chi-square with fisher's exact tests.

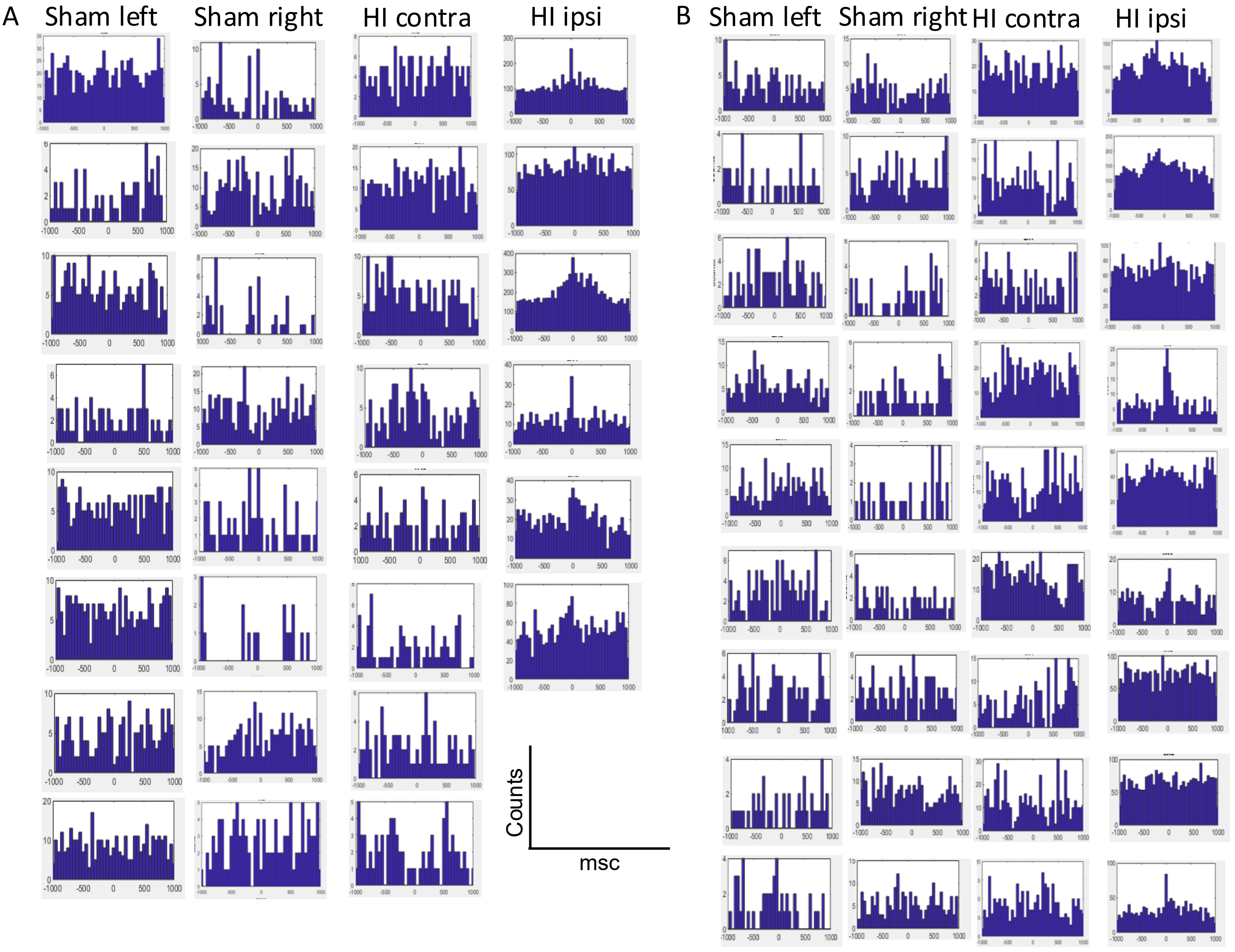

Figure S4 Additional examples of Cross-correlograms of cell pairs in the RSC and hippocampus. Cross-correlograms of cell pairs from the (A) RSC and (B) hippocampus suggest that cell pairs from right and left sham hemispheres and the contralateral HI hemisphere had weakly correlated spike times. The ipsilateral HI hemisphere however, exhibited strong synchronization between neuronal pairs.

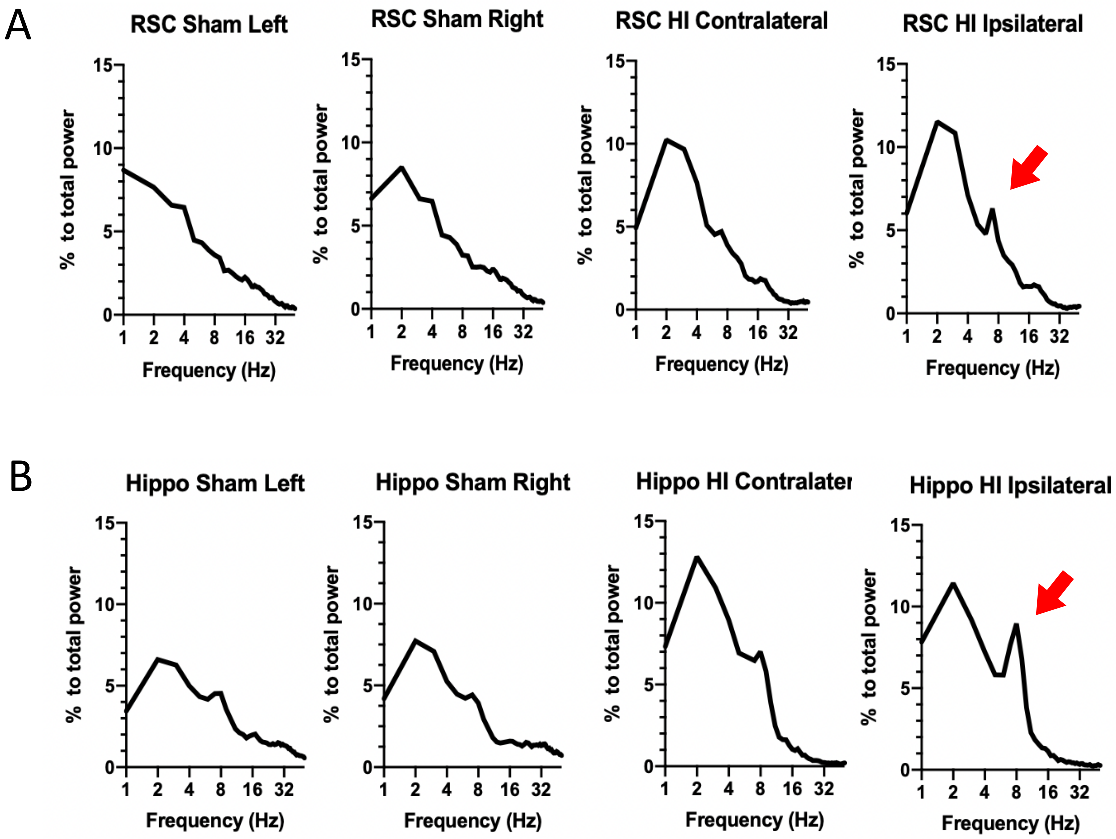

Figure S5 Neonatal HI impairs firing frequency in the cortex and hippocampal neurons. The comparison of (A) RSC and (B) hippocampal field potentials between right and left sham, and contralateral and ipsilateral HI brain waves. The red arrow points to a strong oscillation in the theta band (4-8Hz) which was not observed in sham mice nor the contralateral hemisphere of HI mice.

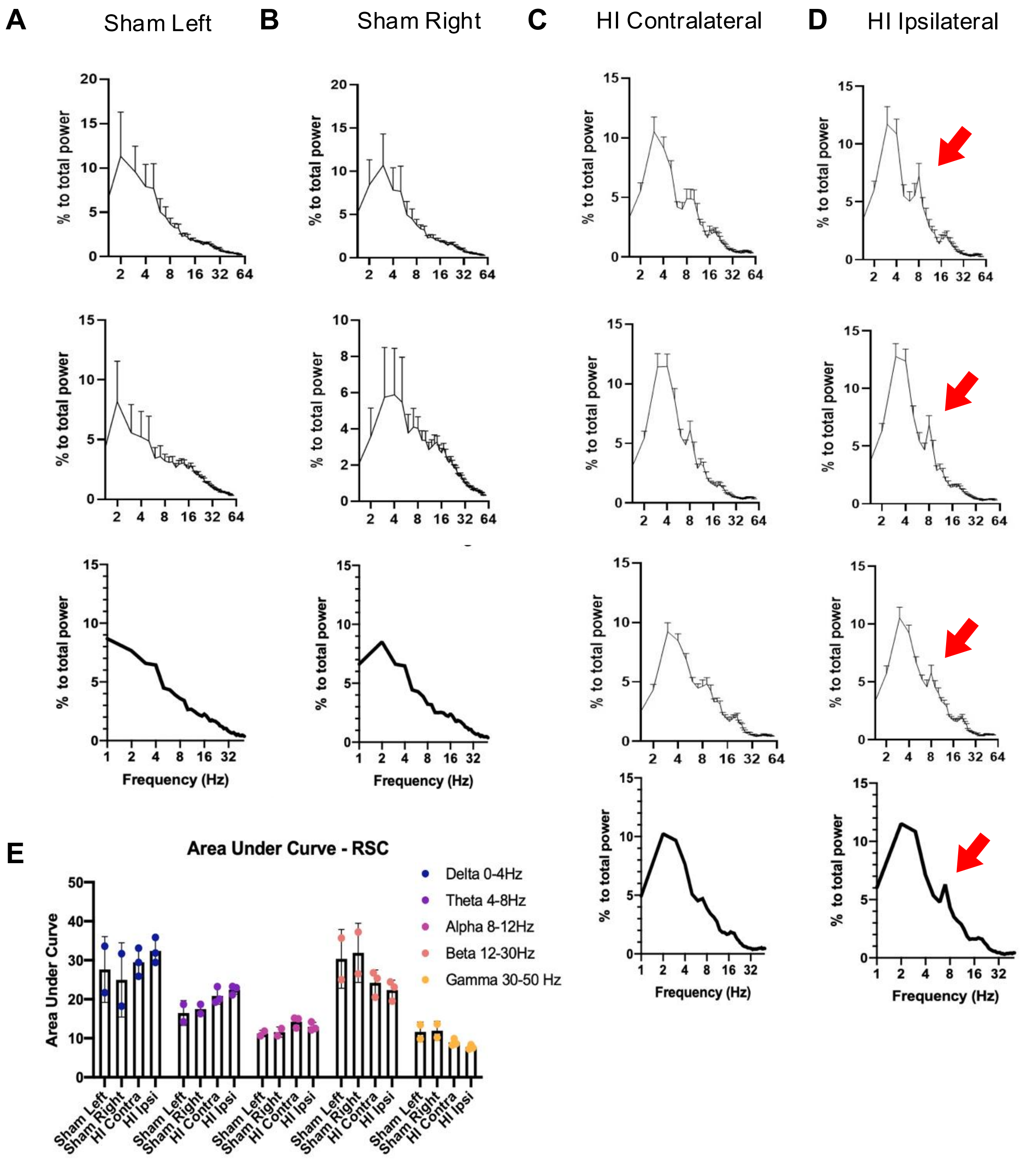

Figure S6 Neonatal HI impairs the firing rate of RSC neurons in individual animals. In all graphs, Log(2) of the field potential was calculated to visualize and identify different peaks. (A-D top row) Power spectra of field potentials in the RSC with all animals combined. (A&B row two and three) Power spectra of field potentials in the left and right RSC of sham1 and sham2 individual mice. (C&D row two, three, and four). Power spectra of field potentials in the contralateral and ipsilateral RSC of HI1, HI2, and HI3 individual mice. Red arrows point to strong oscillations in the Theta band (4-8Hz) in the ipsilateral hemispheres of individual HI mice which was consistent with findings from data with all animals combined. (E) Results of analyses of the area under the curve of each oscillatory band in the power spectra of the RSC. No significance was found between sham and HI groups within each oscillatory band. (Tukey's multiple comparisons test)

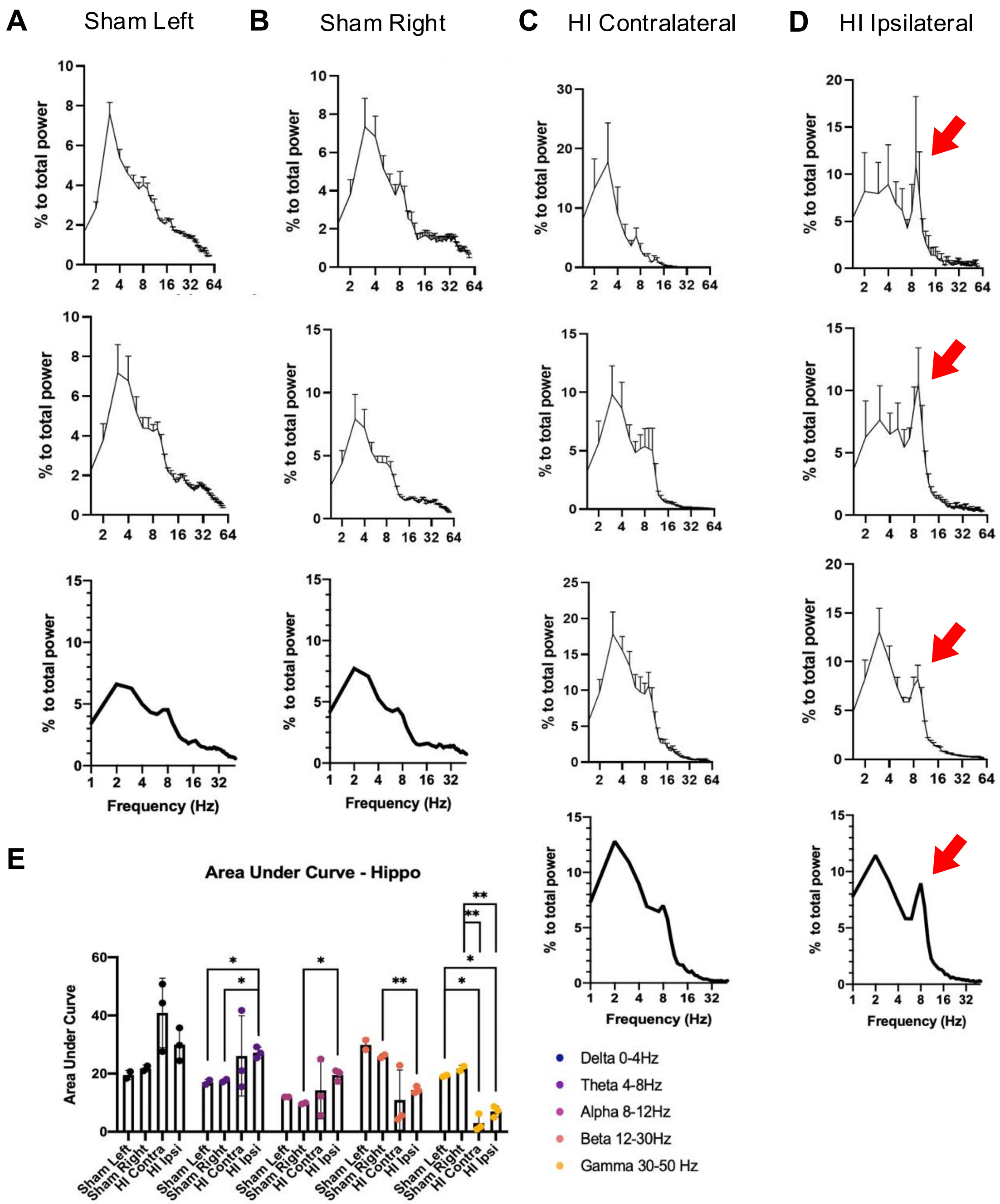

Figure S7 Neonatal HI impairs the firing rate of hippocampal neurons in individual animals. In all graphs, Log(2) of the field potential was calculated to visualize and identify different peaks. (A-D top row) Power spectra of field potentials in the hippocampus with all animals combined. (A&B row two and three) Power spectra of field potentials in the left and right hippocampus of sham1 and sham2 mice. (C&D row two, three, and four) Power spectra of field potentials in the contralateral and ipsilateral hippocampus of HI1, HI2, and HI3 mice. A strong oscillation in the Theta band (4-8Hz) was observed in the ipsilateral hemispheres of HI mice which was consistent with findings from data with all animals combined. (E) Results of analyses of the area under the curve of each oscillatory band in the power spectra of the hippocampus. The ipsilateral hemisphere showed significance in theta, alpha, beta, and gamma bands. (*: $p < 0.05$, **: $p < 0.01$; Tukey's multiple comparisons test)

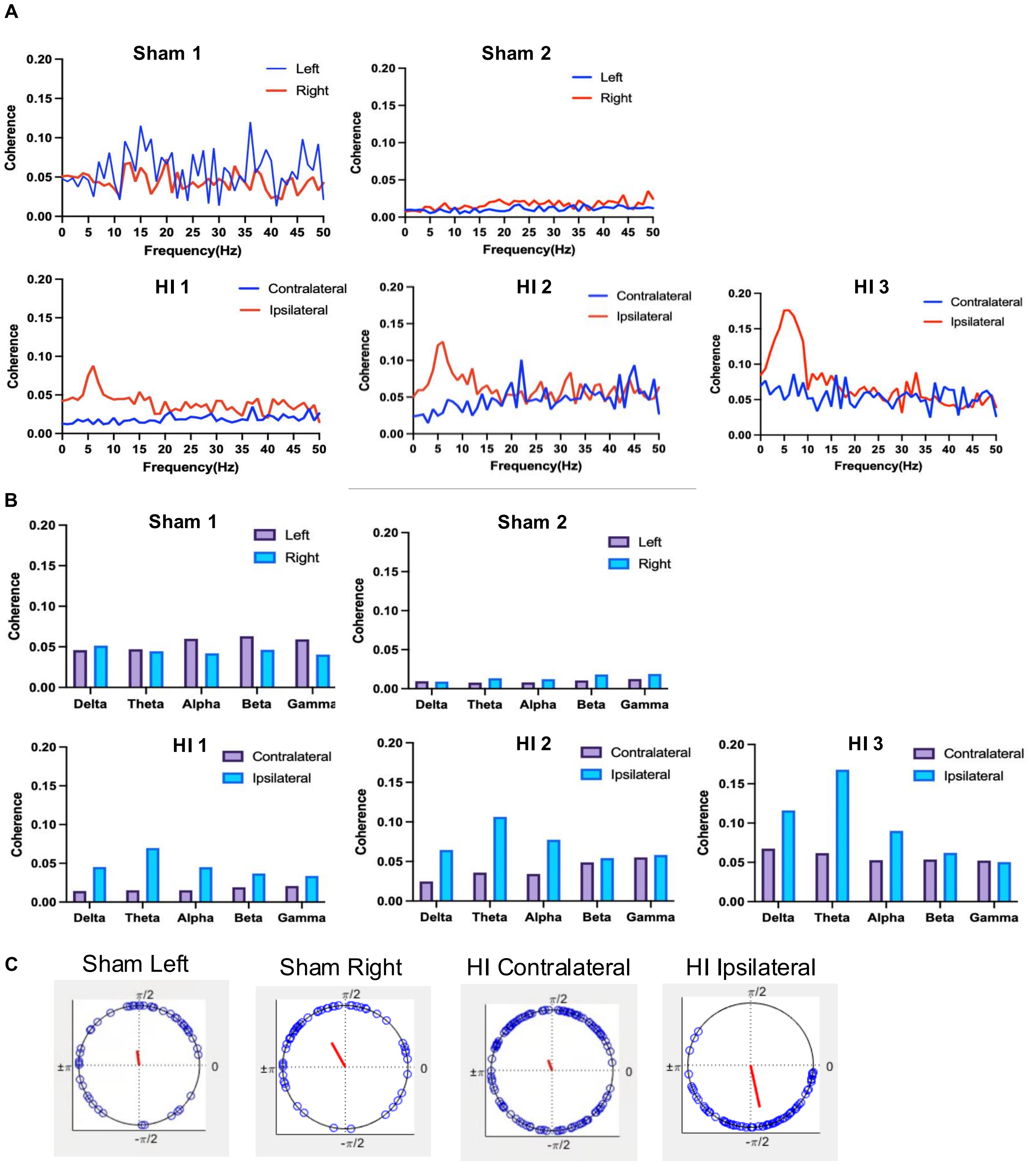

Figure S8 Neonatal HI triggered pathological spike field coherence in the RSC of individual animals. (A) Analysis of the spike-field coherence in the 1-50Hz range showed that the ipsilateral RSC of HI1, HI2, and HI3 mice exhibited a prominent peak in delta, theta and alpha bands (1-12 Hz) compared to sham1 and sham2 mice. (B) Comparison of spike-field activities in the sham RSC's and HI contralateral RSC suggest that RSC PNs in ipsilateral hemispheres of HI1, HI2, and HI3 mice were involved in low frequency (delta-to-alpha band) oscillations. (C) Polar-histogram of the spike-field coherence phase distribution in theta-to-alpha band (4-12 Hz). Phase-locking bias between spikes and LFP was evident in the (C) injured ipsilateral RSC in HI1, HI2, and HI3 mice, which was absent in both the (A) sham1 and sham2 mice and (B) contralateral side of HI1, HI2, and HI3 mice.

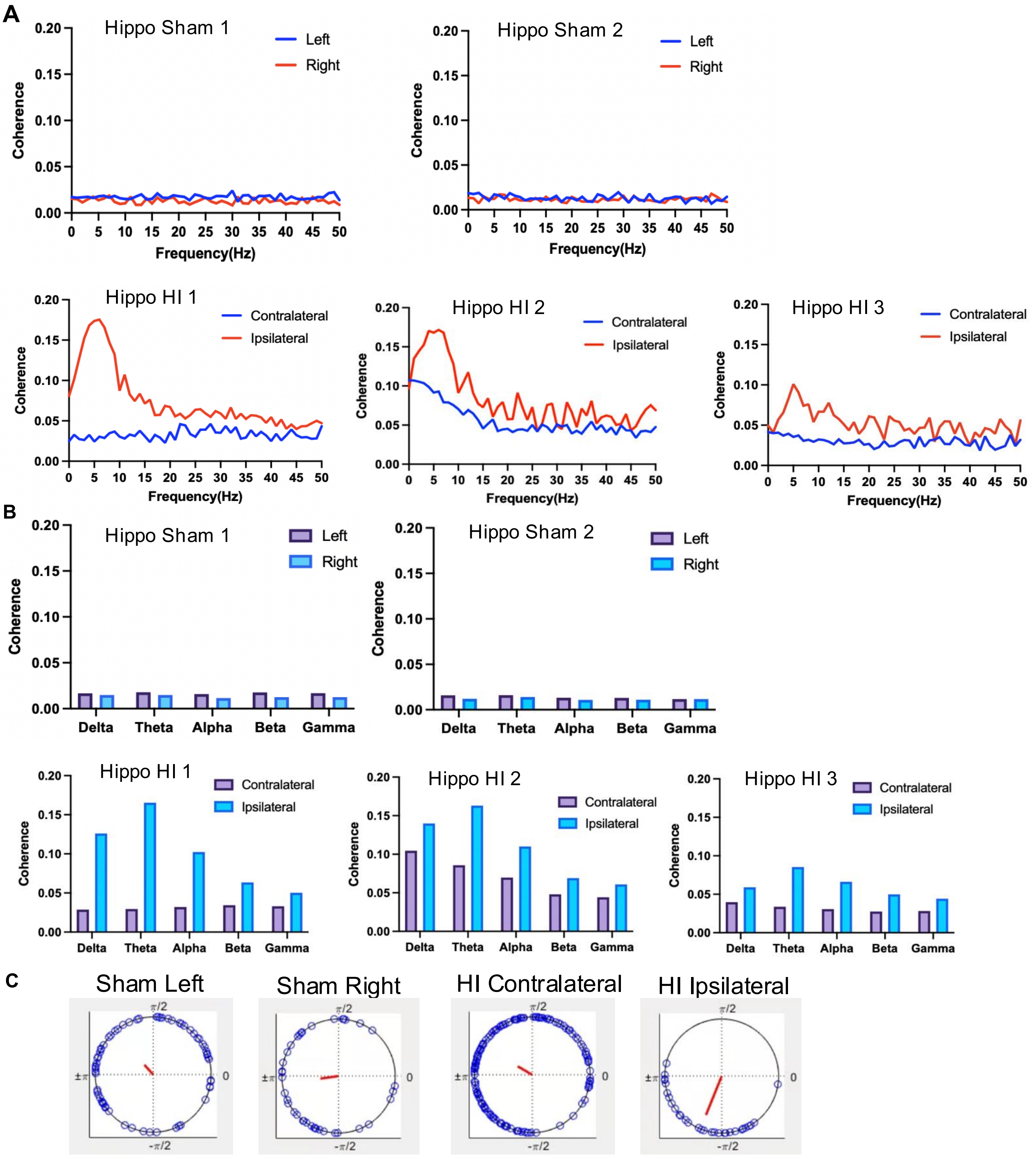

Figure S9 Neonatal HI triggered pathological spike field coherence in the hippocampus of individual animals. (A) Analysis of the spike-field coherence in the 1-50Hz range showed that the ipsilateral hippocampus of HI1, HI2, and HI3 mice exhibited a prominent peak in delta, theta and alpha bands (1-12 Hz) compared to sham1 and sham2 mice. (B) Comparison of spike-field activities in sham hippocampi and the HI contralateral hippocampus suggested that hippocampal PNs in ipsilateral hemispheres of HI1, HI2, and HI3 mice were involved in low frequency (delta-to-alpha band) oscillations.

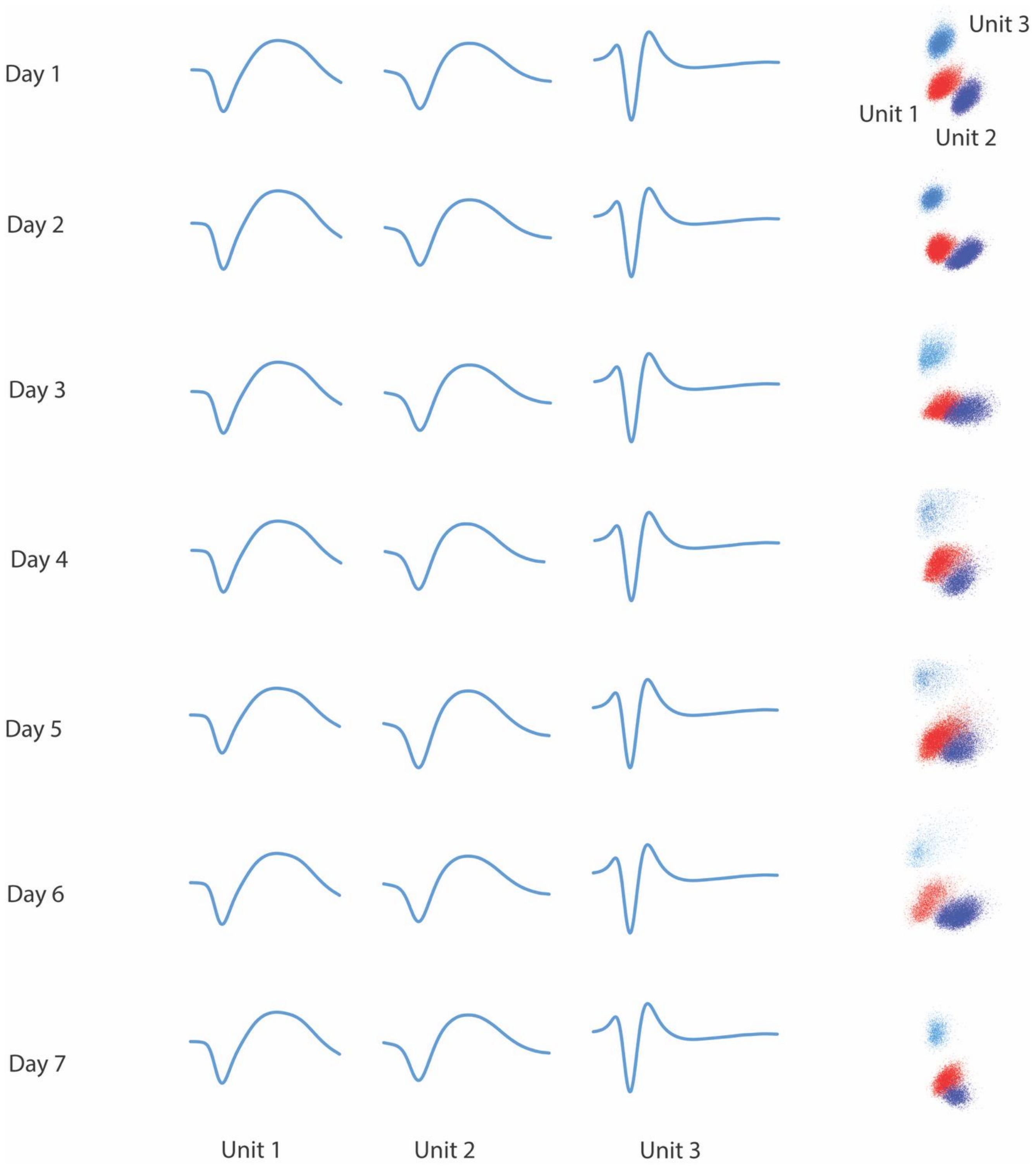

Figure S10 Sample neurons being traced in 7 days of rotarod training. (C) Polar-histogram of the spike-field coherence phase distribution in theta-to-alpha band (4–12 Hz). Phase-locking bias between spikes and LFP was absent in (A) sham1 and sham2 mice and (B) contralateral side of HI1, HI2, and HI3 mice while the (C) injured hippocampus in HI1, HI2, and HI3 mice showed pathological phase-locking bias.
